# Magnetic Graphene Composites: From Rational Synthesis, Structural Design to Multifunctional Applications

**DOI:** 10.3390/molecules31132285

**Published:** 2026-06-30

**Authors:** Yanlong Liang, Pengfei Tian, Wei Wang, Shan Jin, Yun Zhao, Ruyi Li, Guiru Ma, Canliang Ma

**Affiliations:** 1 Shanxi Road & Bridge Qingyin-Erguang Expressway Taiyuan Liaison Line Co., Ltd., Jinzhong 045400, China; lyl11648@163.com (Y.L.); tyustww@163.com (W.W.); jcf314159@163.com (S.J.); 2 Key Laboratory of High-Performance Energy Storage Materials and Systems of Shanxi Province, Institute of Molecular Science, Shanxi University, Taiyuan 030006, China; nounou12319@163.com (P.T.); zhaoyun@sxu.edu.cn (Y.Z.); 19911835193@163.com (R.L.); 15735367203@163.com (G.M.)

**Keywords:** magnetic graphene composites, controllable synthesis, structural engineering, structure-property relationship, multifunctional applications, rational design

## Abstract

Magnetic graphene composites have emerged as a frontier material platform, offering designable properties and multifunctional integration across environmental, biomedical, electromagnetic, and energy applications. Despite extensive research, a coherent knowledge framework that systematically connects synthesis, structure, property, and application remains lacking. This review addresses this gap by establishing an integrated “synthesis–structure–property–application” design paradigm. We first propose a four-tier evolutionary framework for synthesis strategies, tracing the progression from modular in-situ assembly, substrate-guided single-component in-situ formation, and synchronous in-situ formation to molecular-scale precursor co-conversion. This framework reveals the causative relationships between synthesis pathways and microstructures, and culminates in an application-oriented synthesis decision-making tool that enables rational strategy selection. Building on this synthesis foundation, we systematically analyze three core structural regulation strategies—interface engineering, defect and doping engineering, and hierarchical structure construction—demonstrating how they function as synergistic “control knobs” for tailoring composite properties. Through detailed case studies across four application domains, we quantitatively show how targeted structural design drives performance breakthroughs: enabling high-capacity and selective pollutant removal in environmental remediation; constructing intelligent theranostic platforms in biomedicine; reconciling the “thin, lightweight, broadband, and strong” paradox in electromagnetic interference (EMI) shielding; and ensuring long-cycle stability of high-capacity electrodes in energy storage. Finally, we summarize the paradigm shift from “functional combination” to “performance synergy” and outline future directions, including dynamic intelligent systems, sustainable manufacturing, and data-driven design. This review provides a systematic theoretical framework and practical roadmap for the rational design and on-demand fabrication of MGCs, marking the field’s transition from empirical exploration toward predictive, design-driven science.

## 1. Introduction

Graphene, a two-dimensional monolayer of sp^2^-hybridized carbon atoms arranged in a honeycomb lattice, has been hailed as a revolutionary material since its isolation. Its extraordinary physicochemical properties—including an ultra-high theoretical specific surface area (~2630 m^2^/g), exceptional charge carrier mobility (>200,000 cm^2^/V·s), outstanding mechanical strength, and superior thermal conductivity—have unlocked tremendous potential in electronics, optoelectronics, composites, and energy applications [1,2,3,4,5,6]. However, the practical application of pristine graphene is hindered by several intrinsic limitations. First, its zero-bandgap semimetallic nature critically restricts its use in logic semiconductor devices. Second, its inherent diamagnetism fails to meet the urgent demand for magnetically responsive functionalities essential for applications like magnetic separation, targeted delivery, and magnetic resonance imaging (MRI). Third, processing challenges arise from the strong van der Waals forces between sheets, leading to irreversible agglomeration and restacking, which severely compromises the utilization of its immense theoretical surface area and active sites in macroscopic assemblies [7,8]. Fourth, while its chemical inertness ensures stability, it also poses difficulties for specific surface functionalization and effective integration with other materials or biological systems [9,10].

**Figure 1:**
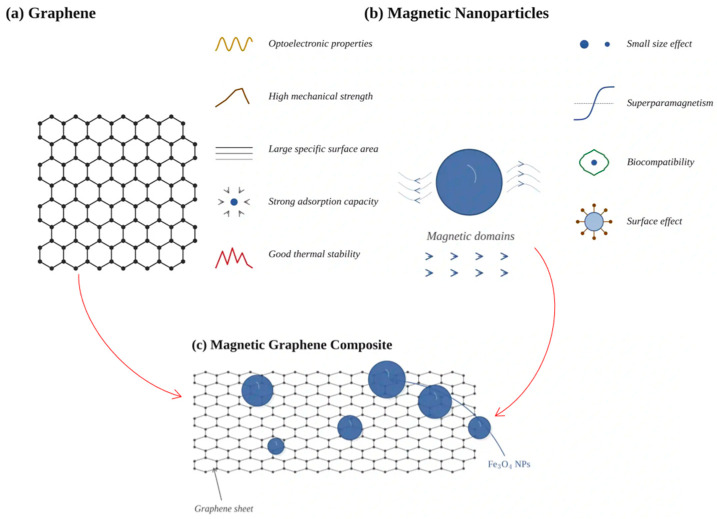
Schematic illustration of the structure of MGCs, depicting the integration of magnetic nanoparticles (e.g., Fe_3_O_4_, γ-Fe_2_O_3_, CoFe_2_O_4_) onto a graphene scaffold. This diagram establishes the foundational binary-system motif that underpins the entire “synthesis-structure-property-application” analysis in this review.

To overcome these bottlenecks, compositing graphene with functional nanounits has emerged as a pivotal strategy. Among these, magnetic graphene composites (MGCs) have been developed forming the representative binary structural archetype of MGCs [11,12,13,14,15,16] by integrating graphene with magnetic nanoparticles (e.g., Fe_3_O_4_, γ-Fe_2_O_3_, CoFe_2_O_4_), as schematically illustrated in Figure 1. The magnetism of these magnetic graphene composites differs from that induced by the structure of graphene itself [17,18,19,20,21,22,23,24]. Their magnetism originates from the doped magnetic nanomaterials. These materials not only inherit the high electrical conductivity and large specific surface area of graphene but also incorporate capabilities—such as facile separation, targeting, actuation, and hyperthermia therapy via external magnetic fields—that are conveniently tunable. These hybrids not only inherit the advantages of individual components but also, through synergistic effects, unlock unprecedented application potential across four key domains: environmental remediation, biomedicine, electromagnetic functional materials, and energy storage and conversion [14,15,25,26,27]. Certainly, magnetic graphene also has applications in other fields, such as sensors [28]. However, due to the limited number of cases, we will not elaborate further here.

Nevertheless, realizing the theoretical potential of Magnetic graphene composites (MGCs) and translating it into practical applications requires systematically addressing two core, interconnected scientific questions: How to achieve the controllable fabrication of these materials (synthesis science)? And how to precisely tailor their macroscopic performance through microscopic structural design (structural engineering)? These questions form a complete chain from “material creation” to “function realization.”

On the synthesis front, despite the proliferation of diverse methods, a fundamental disconnect persists. Existing reviews have predominantly cataloged methods by application or component type [17,18,19,20,21,22,23,24], offering a descriptive taxonomy but failing to capture the underlying evolutionary logic and, more importantly, the causative “synthesis-pathway → microstructure” relationships that dictate final properties. Bridging the gap between synthetic methodology and performance realization is a critical “transducer”: structural engineering. The macroscopic performance of a composite is not a simple sum of its parts but is dictated and amplified by its microstructure. This microstructure—encompassing interfaces, defect/doping states, and hierarchical morphology—functions as a suite of sophisticated “control knobs.” While numerous successful application-specific cases exist, a significant gap remains: the consolidation of cross-domain structural design principles into a systematic, performance-oriented design blueprint and decision-making roadmap.

To address these gaps, this review establishes an integrated “Synthesis-Structure-Property-Application” knowledge framework. We first propose a four-tier evolutionary framework for synthesis strategies, which reveals causative synthesis-structure relationships and culminates in an application-oriented decision-making tool. Building on this, we analyze three core structural regulation paradigms—interface engineering, defect and doping engineering, and hierarchical structure construction—demonstrating how they translate synthetic potential into targeted functionalities. Through case studies across environmental remediation, biomedicine, electromagnetic interference (EMI) shielding, and energy storage, we show how domain-specific structural design overcomes core performance bottlenecks. This work provides a practical roadmap for transitioning from empirical exploration to rational design.

## 2. The Four-Tier Evolution of Synthesis Strategies: From Modular Assembly to Molecular Co-Conversion

The controllable synthesis and performance optimization of high-quality magnetic graphene composites face core challenges that are fundamentally rooted in synthesis science. First, regarding precise structural control, achieving the highly dispersed, uniform, and firmly anchored deposition of magnetic nanoparticles on graphene substrates, while simultaneously tuning the defect and doping states of graphene, is crucial to prevent particle aggregation, oxidative deactivation, and to fully realize synergistic effects [7,25,26,27]. Second, in terms of process and cost, many conventional methods (e.g., multi-step hydrothermal synthesis, complex assembly) are often cumbersome, require harsh conditions, and are costly, hindering scalable production [29,30]. Third, for multifunctional integration, real-world applications frequently demand materials with multiple responsive (e.g., magnetic, optical, pH) and synergistic properties (e.g., adsorption-catalysis, imaging-therapy), imposing higher requirements on the hierarchical structural design and component synergy of the materials [14,31,32,33].

In response to these challenges, synthesis strategies have continuously evolved. The literature indicates a shift in research focus from simple physical blending (e.g., In-situ Assembly, as seen in refs [8,34,35,36,37,38,39,40,41,42,43,44,45,46,47]) towards more integrated chemical construction. For instance, Single-Component In-situ Formation (e.g., [9,26,27,30,48,49,50,51,52,53,54,55,56,57,58,59,60,61,62,63,64,65,66,67,68,69,70,71,72,73,74,75,76,77,78,79,80,81,82,83,84,85,86,87]) and Synchronous In-situ Formation (e.g., [7,29,88,89,90,91,92,93,94,95,96,97,98,99,100,101,102,103,104,105,106,107,108,109,110,111,112,113,114]) enable the simultaneous synthesis of components and their integration in a one-pot reaction, enhancing efficiency and interfacial bonding. The most prospective strategy, Precursor Co-Conversion (e.g., [31,90,91,103,115,116,117]), achieves molecular/atomic-scale integration through the co-conversion of designed precursors (via CVD [31], joule heating [115], or self-catalytic pyrolysis [116,117]). This results in carbon-layer-encapsulated magnetic cores (core-shell structures) and doped graphene matrices, optimizing the interface, electronic structure, and stability at the atomic level. It represents a significant trend towards greener, lower-temperature, and more precise material fabrication.

Despite the proliferation of diverse synthesis routes, a fundamental disconnect persists between the description of methods and the prescription for their rational selection. Existing reviews have predominantly cataloged these methods by application domain (e.g., adsorption, biomedicine) or component type, offering a useful but inherently descriptive and retrospective taxonomy [11,12,13,14,15,16,17]. This approach, while informative, fails to capture the underlying evolutionary logic of the field—a logic defined by the relentless pursuit of higher manufacturing precision, deeper interfacial engineering, and greater process integration. Consequently, a unifying conceptual framework has been conspicuously absent. Such a framework should not only map historical progress but also provide forward-looking guidance for synthesis strategy selection. This gap leaves researchers without a principled roadmap. Consequently, they struggle to navigate the complex trade-offs between performance goals, process complexity, and practical constraints, and often revert to empirical trial-and-error for strategy selection.

**Figure 2:**
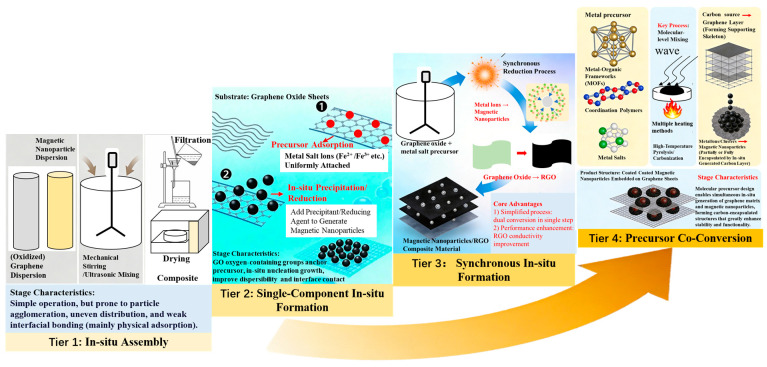
Proposed four-tier evolutionary framework for synthesis strategies of MGCs: (I) In-situ Assembly, (II) Single-Component In-situ Formation, (III) Synchronous In-situ Formation, and (IV) Precursor Co-Conversion. The diagram distills the field’s underlying evolutionary logic—from modular combination to molecular-scale integration—revealing the causative synthesis-pathway to microstructure relationships that previous taxonomies have overlooked.

**Figure 3:**
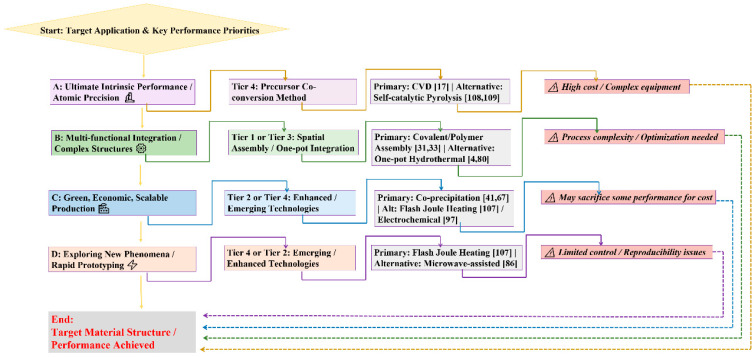
Application-oriented synthesis decision-making framework for MGCs. Translating the mechanistic insights of the four-tier evolutionary framework (Figure 2) into a practical workflow, this flowchart guides researchers to rationally select the optimal synthesis strategy based on performance priorities, resource constraints, and scalability requirements.

To bridge this gap, we propose a four-tier evolutionary framework (Figure 2) that moves beyond descriptive cataloging to reveal the causative synthesis-pathway to microstructure relationships. This framework posits a clear trajectory from modular combination to molecular-scale integration, and our analysis dissects these relationships within each tier, linking chemical mechanisms to material properties. However, a complete synthesis science paradigm must offer not only a lens for understanding the past but also a tool for designing the future. Therefore, the culminating and practical contribution of this work is the translation of this deep mechanistic understanding into a first-of-its-kind, application-oriented synthesis decision-making framework (See Section 3.1 and Figure 3). This framework empowers researchers to move decisively from empirical exploration to rational design by providing a systematic workflow to select the optimal synthesis strategy based on specific performance priorities, resource limitations, and scalability requirements. By detailing this evolutionary logic and its corresponding decision-making tool, this work aims to establish a new, more deliberate, and predictive science of precision synthesis for magnetic graphene composites.

The performance leap of magnetic graphene composites is rooted in the profound evolution of their synthesis strategies, from rudimentary combination to sophisticated integration. As illustrated in Figure 2, this evolutionary path can be clearly categorized into four tiers with progressively increasing complexity and integration: In-situ Assembly, Single-Component In-situ Formation, Synchronous In-situ Formation, and Precursor Co-Conversion. Table 1 systematically compares the core principles, advantages, challenges, and representative literature of these four categories, outlining the overall blueprint of the field’s development. This evolution is not a simple stacking of techniques but reflects a continuous pursuit of higher precision in controlling the material’s interface, component interaction, and final structure, serving as the bridge connecting “controllable fabrication” with “high-performance realization”.

Table 1 crystallizes a fundamental tension that pervades the entire synthesis science of MGCs: the inverse relationship between structural precision and scalability. Tier 1 (In-situ Assembly) offers unmatched modular flexibility and operational simplicity, yet its Achilles’ heel—weak interfacial bonding—limits long-term performance in demanding environments. Tier 4 (Precursor Co-Conversion) delivers atomic-level integration and ultimate stability, but at the cost of harsh conditions and prohibitive expense. The critical insight here is that there is no single “best” method; rather, the optimal choice is inherently context dependent. What the field urgently needs is not another incremental improvement to an existing method, but a systematic framework for navigating these trade-offs—precisely the gap our proposed decision-making tool (Figure 3) aims to fill. Furthermore, we note a conspicuous lack of systematic studies comparing the same material system across multiple tiers under standardized conditions. Such head-to-head comparisons would be invaluable for quantifying the true performance premium of moving up the evolutionary ladder.

### 2.1. Tier 1: In-Situ Assembly—The Starting Point of Modular Construction

In-situ assembly is one of the most classic and intuitive strategies for constructing magnetic graphene composites. This method follows the modular concept of “first independent synthesis, then directed integration”, where the graphene component (Graphene oxide (GO), reduced graphene oxide (RGO), or their functionalized derivatives) and the magnetic nanoparticles (e.g., Fe_3_O_4_ γ-Fe_2_O_3_, CoFe_2_O_4_) are prepared separately, and then assembled into a composite system using physical or chemical interactions [3,19,20,21,22,23,24,25,26,27,28,29,30,31,32]. The general procedure, as shown in Figure 2, involves mixing a GO dispersion with a magnetic nanoparticle dispersion via mechanical stirring or ultrasonication, utilizing the intermolecular forces (e.g., electrostatic attraction, covalent bonding, van der Waals forces) to achieve combination, followed by separation and drying to obtain the composite powder.

The advantage of this strategy lies in its clear synthetic pathway and high process flexibility. The pathway allows for independent pre-optimization of each component, such as controlling the size and crystallinity of magnetic particles, or specifically functionalizing GO. It is particularly suitable for constructing multi-level architectures with distinct functional partitions. However, its core challenge is overcoming the relatively weak interfacial interactions to achieve uniform and stable loading of magnetic particles on the graphene substrate, preventing performance degradation due to agglomeration or desorption. Based on the nature of the driving forces for assembly, in-situ assembly can be mainly divided into three categories: electrostatic self-assembly, covalent bonding assembly, and polymer/biomolecular bridging, which differ significantly in interfacial bond strength, process complexity, and functional tunability.

#### 2.1.1. Electrostatic Self-Assembly

Electrostatic self-assembly utilizes the Coulombic attraction between negatively charged GO (whose surface carboxyl and hydroxyl groups ionize in solution) and magnetic nanoparticles modified to carry an opposite surface charge, driving their spontaneous adsorption and combination [3,25]. For instance, Fe_3_O_4_ particles synthesized via the common co-precipitation method are typically rich in surface hydroxyl groups, which results in an electrically neutral or slightly negative surface charge. They are often modified with cationic surfactants (e.g., cetyltrimethylammonium bromide, CTAB) to impart a positive charge, enabling effective combination with negatively charged GO via electrostatic attraction. Zhu et al. [3] employed this strategy to assemble CTAB-modified Fe_3_O_4_ nanoparticles onto a three-dimensional graphene foam skeleton, fabricating a composite with both flexibility and high electrical conductivity, demonstrating excellent electromagnetic interference shielding performance.

The prominent advantages of this method are its rapid process and mild conditions, requiring no complex chemical reactions. However, the physical adsorption force it relies on is relatively weak, resulting in insufficient interfacial bond strength. Moreover, the assembly process is highly sensitive to the solution environment (e.g., pH, ionic strength) and is prone to dissociation in complex practical application systems, potentially affecting the long-term stability and recyclability of the material.

#### 2.1.2. Covalent Bonding Assembly

To overcome the weak interfacial force of electrostatic assembly, the covalent bonding assembly strategy is widely adopted. This method involves constructing covalent chemical bonds (primarily amide bonds) between the oxygen-containing functional groups on GO (mainly -COOH) and the active groups (e.g., -NH_2_) on the surface-modified magnetic nanoparticles, achieving a robust connection between the two components [45,117]. This process typically employs carbodiimide coupling agents (e.g., 1-ethyl-3-(3-dimethylaminopropyl) carbodiimide, EDC) and activators (e.g., N-hydroxysuccinimide, NHS). EDC/NHS first activates the carboxyl groups on GO to form a reactive intermediate, which then undergoes nucleophilic substitution with the amino groups on the surface of magnetic particles modified with aminosilanes (e.g., 3-aminopropyltriethoxysilane, APTES), forming stable amide bonds (-CO-NH-).

Gonzalez-Rodriguez et al. [45] used this strategy to covalently graft APTES-modified Fe_3_O_4_ nanoparticles onto GO, constructing a multifunctional theranostic platform integrating magnetic targeting, drug delivery, and MRI/fluorescence dual-modal imaging. In the field of environmental catalysis, Zhao et al. [118] employed similar covalent assembly to integrate amine-functionalized Fe_3_O_4_ cores and MIL-101(Fe) shells on GO surfaces, constructing a core-shell composite material successfully used for activating peroxymonosulfate in advanced oxidation processes to degrade organic pesticides.

Covalent bonding assembly provides one of the strongest interfacial connections, enabling the composite material to maintain excellent structural integrity in harsh physicochemical environments (e.g., in vivo, catalytic reaction systems), making it suitable for applications requiring long-term cycling or high stability. Its limitations include relatively cumbersome steps, the use of toxic coupling agents, and the need for precise control of reaction conditions (pH, temperature, time) to ensure high grafting efficiency and reproducibility.

#### 2.1.3. Polymer/Biomolecular Bridging and Functionalized Assembly

This strategy introduces functional polymers, biomacromolecules, or functional monomers as “linkers” or “interfacial layers” to achieve assembly through various non-covalent/covalent interactions (e.g., hydrogen bonding, coordination, coating, in-situ polymerization), while simultaneously endowing the composite with enhanced dispersibility, excellent biocompatibility, stimuli-responsiveness, or specific recognition capabilities [42,46,47,119].

(1) Polymer Coating/Bridging: Utilizes natural or synthetic polymers to connect the two components via physical coating or chemical crosslinking. For example, Ramezani Farani et al. [42] co-coated pre-synthesized GO-Fe_3_O_4_ with biocompatible chitosan (CS) and polyvinyl alcohol (PVA) through simple solution mixing and vacuum drying, significantly improving its aqueous dispersibility for loading and release of the model drug 5-fluorouracil. Cui et al. [47] developed a more sophisticated strategy, synthesizing folic acid (FA)-targeted thiolated chitosan, which was crosslinked in one step with thiolated GO (TGO) under sonication to form nanocapsules possessing magnetic targeting and pH/reduction dual-responsive release capabilities.

(2) Biomolecule Modification: Wate et al. [46] designed a multifunctional fluorescent probe, first covalently anchoring polyamidoamine dendrimers (PAMAM G4) to GO, then using the bifunctional molecule glutathione (GSH) as a bridge to covalently link Fe_3_O_4_ nanoparticles, and finally, labeling with the near-infrared fluorescent dye Cy5, achieving efficient fluorescent imaging of tumor cells.

(3) In-situ Polymerization of Functional Monomers: Liu et al. [119] grafted a polyamidoxime/polyethylenimine (PAO/PEI) polymer chain rich in amidoxime and amine groups onto the surface of magnetic graphene oxide (MGO) via in-situ polymerization, greatly enhancing the composite’s adsorption capacity, selectivity, and anti-interference ability towards the radioactive nuclide uranyl ion U(VI).

Polymer/biomolecular bridging significantly expands the functional dimensions of composite materials and is a key technical pathway for achieving active targeting, controlled release, high bio-affinity, or specific pollutant recognition. However, the introduction of bridging molecules may partially cover the active sites on the GO surface, and the multi-step, complex modification process can pose challenges for the reproducibility of batch production.

#### 2.1.4. Methodological Comparison and Evolutionary Context

To clearly contrast the intrinsic differences and application orientations of the three in-situ assembly strategies mentioned above, their core characteristics are summarized in Table 2. From electrostatic self-assembly to covalent bonding assembly, and then to polymer/biomolecular bridging, the dominant forces transition from physical to combined chemical and physical interactions, with corresponding increases in interfacial bond strength and process complexity. This directly determines material stability: electrostatically assembled materials are sensitive to the environment, covalently bonded materials offer the highest stability, and polymer-bridged materials can achieve a balance between stability and functional flexibility through clever molecular design. Therefore, these three strategies are respectively suited for different performance requirements and application scenarios: electrostatic self-assembly is suitable for occasions requiring rapid combination without demanding strong bonding forces (e.g., electromagnetic shielding, basic adsorption); covalent bonding assembly is the foundation for constructing functional platforms that need to operate stably in harsh environments over the long term (e.g., biotheranostics, advanced oxidation catalysis); and polymer/biomolecular bridging specializes in high-end applications requiring excellent biocompatibility, active targeting, stimuli-responsiveness, or ultra-high selectivity (e.g., targeted drug delivery, specific separation). This comparison profoundly reveals the inherent “performance-cost-complexity” trade-offs within in-situ assembly methods, providing a basis for their rational selection.

Table 2 illuminates a clear gradient from physical to chemical bonding, with corresponding gains in interfacial strength and stability. However, what the table cannot capture is the often-overlooked cost of “over-engineering” the interface. Covalent bonding assembly, while offering the strongest connection, introduces multiple chemical modification steps that can compromise batch-to-batch reproducibility—a critical concern for translational applications. Polymer/biomolecule bridging, despite its functional versatility, risks passivating the graphene surface and blocking active sites. Our perspective is that the choice of assembly strategy should be guided not by a blanket preference for the “strongest” bond, but by a careful assessment of the operational environment: electrostatic assembly may be perfectly adequate for one-time adsorption applications in benign conditions, while covalent bonding becomes non-negotiable for long-term in vivo biomedical devices. The field lacks systematic guidelines for making this “fit-for-purpose” decision, which our framework begins to address.

### 2.2. Tier 2: Single-Component In-Situ Formation—Substrate-Guided Chemical Anchoring

The Single-Component In-situ Formation method refers to strategies where pre-prepared graphene oxide (GO) or functionalized graphene serves as a stable substrate and precursor. Magnetic metal ions (e.g., Fe^3+^, Fe^2+^, Co^2+^) are directly converted and loaded in-situ into nanoparticles anchored onto this substrate surface through chemical reactions such as hydrothermal/solvothermal reduction, chemical co-precipitation, or microwave-assisted synthesis [9,26,27,29,30,48,49,50,51,52,53,54,55,56,57,58,59,60,61,62,63,64,65,66,67,68,69,70,71,72,73,74,75,76,77,78,79,80,81,82,83,84,85,86,87]. The core of this method lies in the simultaneous completion of magnetic active phase formation and the “functionalization” of the graphene matrix within the same reaction system, achieving chemical bonding between the particles and the substrate. Compared to simple physical mixing, this method typically yields composites with more uniform dispersion and stronger interfacial bonding. The general process usually starts with GO prepared via the modified Hummers method. This material is rich in carboxyl, hydroxyl, and epoxy groups, providing active sites and dispersion stability for subsequent reactions. Based on differences in reaction systems and driving forces, this method mainly gave rise to subclasses with distinct characteristics: hydrothermal/solvothermal, co-precipitation, and microwave-assisted methods.

#### 2.2.1. Hydrothermal/Solvothermal Method

The hydrothermal/solvothermal method utilizes a sealed high-temperature, high-pressure reaction environment to synchronously drive the thermal reduction of GO and the nucleation/crystallization growth of magnetic nanocrystals. It is an effective means of constructing nanocomposites with well-defined structures and high crystallinity. A dispersion of GO is uniformly mixed with a stoichiometric amount of magnetic metal salts (e.g., FeCl_3_, Co(NO_3_)_2_), transferred to a Teflon-lined autoclave, and reacted at 120–200 °C for several hours. During this process, GO is partially thermally reduced, decreasing its oxygen-containing functional groups and enhancing conductivity. Simultaneously, metal ions hydrolyze, condense, and grow along specific crystal planes, forming well-crystallized magnetic oxide nanoparticles (e.g., Fe_3_O_4_, CoFe_2_O_4_), which anchor onto graphene sheets via chemical interactions. This method enables:

(1) Complex Structure Construction and Performance Optimization: G. Bharath et al. [26] mixed porous graphene with an iron salt solution, adjusted the pH with ammonia, and reacted hydrothermally at 180 °C for 12 h, successfully generating and loading uniformly distributed Fe_3_O_4_ nanoparticles in-situ within the skeleton and pores of porous graphene. Zhang et al. [48] used ingenious solvothermal design to synchronously synthesize Cu/Fe_3_O_4_ heterogeneous nanospheres in-situ on defect-rich graphene (GE-N), constructing a hierarchical structure with multi-interface loss mechanisms for efficient electromagnetic wave absorption.

(2) Core-Shell and Hierarchical Structure Design: Liu et al. [43] used ferrocene as both an iron and carbon source. Through a solvothermal method followed by annealing, they grew Fe_3_O_4_@C core-shell microspheres with a unique “pitaya-like” morphology in-situ on rGO surfaces, forming a high-performance electromagnetic wave absorbing composite. Congzhi Fu et al. [50] used a two-step method, first synthesizing Fe_3_O_4_@SiO_2_@TiO_2_-Co core-shell powder, then loading it onto rGO via hydrothermal reaction, constructing a quaternary composite for photocatalytic dye degradation.

(3) 3D Macroscopic Assembly: Research [114] demonstrated a strategy combining solvothermal synthesis with freeze-drying. Using urea as a reducing agent and nitrogen source, GO was reduced to N-doped rGO (N-rGO) and self-assembled into a 3D hydrogel during the reaction, while simultaneously encapsulating CoFe_2_O_4_ nanoparticles. After freeze-drying, a CoFe_2_O_4_/N_-_rGO aerogel with excellent porous structure and electromagnetic functionality was obtained.

The hydrothermal/solvothermal method excels at producing composites that exhibit high crystallinity, controllable morphology and composition, and strong interfacial bonding. Limitations include the need for high-pressure equipment, long reaction cycles, and the challenge of precise control due to the coupling between GO reduction degree and particle growth kinetics.

#### 2.2.2. Co-Precipitation Method

The co-precipitation method involves depositing magnetic nanoparticles directly onto GO surfaces in an aqueous environment containing GO by adding a precipitating agent to cause co-precipitation of magnetic metal ions. This method is mild, simple, low-cost, and the most commonly used strategy for preparing magnetic composites for adsorption and separation. A typical process, as described by Deng et al. [49,120], involves: dispersing GO in a mixed salt solution of Fe^2+^/Fe^3+^ (typically with a molar ratio of 1:2), stirring under an inert atmosphere, slowly adding an alkali like ammonia to adjust the pH to 9–11, and aging the reaction at a certain temperature (e.g., 85 °C). Fe_3_O_4_ nanoparticles are formed via co-precipitation of Fe^2+^ and Fe^3+^ in solution and deposit onto the negatively charged GO sheets. By introducing a reducing agent (e.g., hydrazine hydrate), simultaneous reduction of GO can be achieved to obtain magnetic reduced graphene oxide (M-rGO) [121]. This method is widely used in environmental remediation to prepare high-performance magnetic adsorbents, primarily targeting heavy metals and dyes. MGO prepared by co-precipitation by Deng et al. [49] can effectively and simultaneously adsorb Cd(II) and organic dyes in water. TEM characterization shows that Fe_3_O_4_ particles with a size of about 10–15 nm are uniformly distributed on the wrinkled surface of GO, forming a sandwich-like structure, with a Zeta potential of about −35 mV (pH = 6). The combination of co-precipitation with surface functionalization can substantially increase the material’s affinity for specific pollutants. A series of studies have introduced functional molecules such as ethylenediaminetetraacetic acid (EDTA) [74], polyethylenimine (PEI) [75], and β-cyclodextrin [76] onto MGO, significantly enhancing specific adsorption capacity for Pb(II), Cu(II), U(VI) [69,70], antibiotics [78,117], etc. The greatest advantage of this method is its simple, fast, economical operation, and ease of scale-up. Its main limitations are the broad size distribution of the resulting nanoparticles, their tendency to agglomerate, and the limited reduction degree of GO under mild conditions, which may affect the composite’s conductivity.

#### 2.2.3. Microwave-Assisted Method

In summary, hydrothermal/solvothermal, co-precipitation, and microwave-assisted methods constitute the main body of the Single-Component In-situ Formation strategy. To systematically delineate the application boundaries of these three mainstream technical pathways, a comparison of their core process attributes, product characteristics, and application orientations is summarized in Table 3. This comparison clearly reveals that the stringency of the reaction driving force and conditions directly dictates the structural order and performance ceiling of the product, while also correlating with the economic viability and scalability of the process. Hydrothermal/solvothermal methods, driven by reactions under high temperature and pressure, yield composites with high crystallinity and controllable morphology, making them particularly suitable for fields demanding ultimate intrinsic material performance, such as high-performance electromagnetic wave absorption, catalysis, and highly precise biomedical theranostic platforms [49,50,53]. In contrast, the co-precipitation method, based on mild and rapid solution chemistry, boasts the greatest advantages in process simplicity, low cost, and ease of scaling up, rendering it the preferred choice for preparing large batches of adsorbents or basic functional materials [49,53]. The microwave-assisted method, as an efficient and energy-saving intensification technique, leverages its unique volumetric heating mechanism to exhibit distinctive strengths in synthesis speed and product homogeneity, making it suitable for scenarios requiring rapid synthesis or exploration of novel material structures [12,79]. Consequently, the comparison presented in Table 3 extends beyond a mere listing of technical parameters; it serves as a comprehensive “Method-Performance-Cost” selection guide tailored to different application targets and production conditions. It provides clear guidance: for the pursuit of high performance and structural precision, hydrothermal/solvothermal methods are advisable; when prioritizing economic efficiency and large-scale production, co-precipitation is preferred; and for rapid synthesis or the quest for new material architectures, the microwave-assisted method proves to be a potent tool.

Table 3 reveals a striking methodological trichotomy, where each subclass occupies a distinct niche defined by the interplay of thermodynamic driving force and kinetic control. Hydrothermal/solvothermal methods excel in delivering high crystallinity and morphological diversity, but their prolonged reaction times (typically 12–24 h) and batch-mode operation pose fundamental barriers to industrial translation. Co-precipitation, while elegantly simple and scalable, produces materials with broader particle size distributions and limited GO reduction—a compromise that may be acceptable for adsorption but detrimental for applications requiring high electrical conductivity (e.g., electromagnetic shielding, battery anodes). Microwave-assisted methods offer a tantalizing glimpse of rapid, energy-efficient synthesis, yet their adoption remains limited by the requirement for precursors with suitable dielectric properties. A sobering observation from our survey is that fewer than 5% of studies employing these methods report any systematic optimization of reaction parameters (e.g., temperature ramping profiles, precursor addition rates). The field’s maturation demands a shift from “method demonstration” to “process optimization,” leveraging design-of-experiments and in-situ characterization to establish robust synthesis-structure correlations.

Therefore, the Single-Component In-situ Formation method has laid the foundation for the controllable synthesis of magnetic graphene composites. By flexibly utilizing different reaction modes, effective regulation of material composition, morphology, and interfacial properties can be achieved, meeting the diverse demands across fields ranging from environmental remediation and energy storage to biomedicine. Future development will focus on deepening the mechanistic understanding of these reactions to enable precise design, and on promoting the integration of this method with advanced strategies such as defect engineering and heteroatom doping, in order to create novel materials with superior performance.

### 2.3. Tier 3: Synchronous In-Situ Formation—Integrated “One-Pot” Synthesis

The Synchronous In-situ Formation method, often termed the “one-pot” synthesis, represents a more advanced and highly integrated stage in the fabrication of magnetic graphene composites. Its defining feature is the concurrent realization of GO reduction (forming RGO) and the nucleation, growth, and firm loading of magnetic nanoparticles within a single reaction system containing the graphene precursor (typically GO), magnetic metal sources, and necessary reducing or structure-directing agents [7,29,89,90,91,92,93,94,95,96,97,98,99,100,101,102,103,104,105,106,107,108,109,110,111,112,113,114,115,116]. Compared to strategies involving “independent preparation followed by compositing” or “growth on a pre-formed substrate,” this method breaks the boundaries of stepwise synthesis, enabling the direct and efficient transformation from molecular precursors to the final composite architecture. This not only simplifies the process, reducing energy consumption and cost, but also typically yields a more ideal structure characterized by strong interfacial bonding, uniform particle dispersion, and an optimized RGO conductive network, owing to the intimate chemical interaction established between the magnetic particles and the nascent reduced graphene oxide (RGO) during their simultaneous formation.

#### 2.3.1. Hydrothermal/Solvothermal One-Pot Method: The Mainstream Pathway

The hydrothermal/solvothermal one-pot method is the most prevalent and classic strategy within synchronous in-situ formation. It leverages a single sealed reaction environment to drive both GO reduction and magnetic particle synthesis concurrently. A typical process involves uniformly mixing a GO dispersion, magnetic metal salts (e.g., FeCl_3_, Co(NO_3_)_2_), and reducing agents (e.g., ascorbic acid, hydrazine hydrate, ethylenediamine) or mineralizing agents (e.g., urea, sodium acetate). Under high-temperature, high-pressure conditions (typically 120–200 °C), the reducing agent facilitates the partial reduction of GO to RGO. Concurrently, the metal ions are reduced or hydrolyzed. These species then nucleate and crystallize in-situ, forming magnetic nanoparticles (e.g., Fe_3_O_4_, CoFe_2_O_4_) that anchor onto the newly formed RGO sheets. This process is often accompanied by the self-assembly of a three-dimensional gel network. A representative one-step hydrothermal method reported by Lü Yan’gen’s group involves reacting a mixture of GO, FeCl_3_·6H2O, and ethylenediamine at 180 °C. The reducing and complexing action of ethylenediamine drives the synchronous generation of Fe_3_O_4_ nanoparticles and the reduction of GO. The reduced GO simultaneously self-assembles into a three-dimensional network. After freeze-drying, this process yields a lightweight and porous Fe_3_O_4_/graphene aerogel (Fe_3_O_4_/GA) [123]. Cao et al. [89] employed a similar one-pot hydrothermal approach with ascorbic acid as the reductant, reacting GO with FeCl_3_ to generate and load Fe_3_O_4_ nanoclusters in-situ onto RGO, producing a magnetic composite film suitable for electromagnetic shielding. The work of Liu et al. [81] presented a more innovative approach. Using K_2_FeO_4_ as a single source material that acts both as an oxidant (converting graphite to GO) and an iron source (generating Fe^2+^/Fe^3+^), they significantly simplified the precursor preparation steps and efficiently obtained GO-Fe_3_O_4_ hybrid materials.

#### 2.3.2. Core Case Study: The Decisive Influence of Synthesis Pathway

Research by the Xiu-Juan Li group [9] provides a profound illustration of the core tenet that “the method determines the structure, and the structure dictates the performance.” They systematically compared three distinct one-pot synthesis routes for Fe_3_O_4_/RGO composites: a solvothermal method (MRGO-1 using diethylene glycol, DEG), a hydrothermal method (MRGO-2), and a co-precipitation-chemical reduction method (MRGO-3). Their study demonstrated that subtle differences in the synthesis path—primarily the solvent/reductant system and reaction kinetics—significantly alter the material’s microstructure, surface chemistry, and macroscopic performance.

MRGO-1 (Solvothermal): The use of DEG as solvent and reductant under solvothermal conditions resulted in the most uniform dispersion of Fe_3_O_4_ nanoparticles on RGO sheets and the highest specific surface area. This optimal structure translated to the best adsorption performance for model pollutants.

MRGO-2 (Hydrothermal): While employing a greener aqueous system, this method led to slightly agglomerated nanoparticles, resulting in a lower surface area and subsequently reduced adsorption capacity compared to MRGO-1.

MRGO-3 (Co-precipitation-Chemical Reduction): This two-step aqueous route yielded a composite with a positively charged surface due to the specific adsorption of ions during co-precipitation. This unique surface property endowed MRGO-3 with exceptional selectivity for anionic pollutants like Cr(VI), a performance distinct from the other two materials.

This comparative study is pivotal as it quantitatively validates that the choice of synthesis pathway is not merely procedural but fundamentally engineers the material’s interfacial properties and functionality. It provides crucial, application-specific guidance: select solvothermal routes for high-surface-area adsorbents and it considers co-precipitation-chemical reduction for targeting anionic contaminants.

#### 2.3.3. Methodological Summary and Evolutionary Context

Through a high degree of process integration, the Synchronous In-situ Formation method has advanced the preparation of magnetic graphene composites to a stage characterized by enhanced efficiency and greener processing. It strives to achieve chemical bonding and structural interlocking between components at the very genesis of material formation, thereby endowing the composites with superior intrinsic properties. The aforementioned comparative study [9] underscores that nuanced variations in the synthesis route can have a decisive impact on the final material’s performance profile. This necessitates that researchers adopt an integrated “synthesis-pathway → microstructure → surface-property → application-performance” mindset during material design. A deeper mechanistic understanding of reaction kinetics, coupled with the adoption of advanced reactors like continuous flow systems, will be key. This progress promises to unlock the full potential of synchronous in-situ formation for achieving tailored material performance and advancing scalable, reproducible, and intelligent manufacturing.

### 2.4. Tier 4: Precursor Co-Conversion—Molecular-Scale Precision Integration

The Precursor Co-Conversion method represents the most advanced and sophisticated strategy for achieving deep integration and structural monolithicity of magnetic graphene composites at the molecular/atomic scale. It fundamentally transcends the paradigms of “substrate loading” or “synchronous generation” inherent in previous tiers. Its core principle involves the chemical integration of a magnetic metal source and a carbon/nitrogen source into a single precursor (e.g., metal-organic complexes, coordination polymers, or specific mixtures). Subsequent treatment with an energy input—such as heat, electricity, or a rapid thermal pulse—triggers a co-transformation process encompassing pyrolysis, decomposition, rearrangement, and graphitization. This results in the synchronous generation of (often doped) graphene and highly dispersed magnetic nanoparticles (e.g., Fe_3_O_4_, FexC, Co), which are frequently encapsulated by a graphitic carbon layer [31,90,92,103,115,116,117]. This approach enables the direct, intrinsic conversion from designed molecular precursors to functional nanocomposites. The products are characterized by atomically dispersed active sites, robust carbon-shell protection, precise heteroatom doping, and excellent electronic structure tunability, signifying the forefront of material synthesis towards ultimate precision and high-end applications. Based on the energy input and conversion mode, this method primarily encompasses Chemical Vapor Deposition (CVD), High-Temperature Catalytic Pyrolysis, and emerging rapid conversion technologies (e.g., flash Joule heating, electrochemical synthesis).

#### 2.4.1. Chemical Vapor Deposition (CVD): Vapor-Phase Atomic Epitaxy

Chemical Vapor Deposition facilitates the synchronous vapor-phase epitaxial growth of graphene and magnetic species via the catalytic cracking of gaseous precursors on a substrate, establishing itself as the benchmark technique for producing high-quality, structurally well-defined core-shell nanostructures with near-atomic precision. Typically, a volatile metal-organic compound (e.g., ferrocene) serves as the precursor. At high temperatures (>800 °C) under a carrier gas (Ar/H_2_) atmosphere, it undergoes thermal decomposition on a catalytic substrate (e.g., copper foil). The metal and carbon species separate, migrate, and nucleate. By meticulously controlling parameters, such as temperature, gas flow rate, and duration, the process can be tuned to prioritize graphene layer formation, followed by the aggregation of metal and carbon species on the graphene surface to form magnetic nanoparticles encapsulated by few-layer graphene. The work of Alahmadi et al. [31] stands as a paradigm of the CVD method. Using ferrocene as a single precursor on a copper foil at 950 °C and by precisely modulating the reaction kinetics, they achieved exceptional control: following ferrocene decomposition, carbon species first formed a continuous graphene layer; subsequently, iron atoms and carbon fragments diffused and coalesced on the graphene surface, ultimately forming uniform core-shell nanoparticles with Fe_3_C as the core and multi-layer graphene as the shell (Fe_3_C@graphene). This method provides an efficient and highly controllable route for the synthesis of uniformly encapsulated magnetic nanoparticles. The paramount advantages of CVD include the ability to produce core-shell structures with exceptional crystallinity, minimal defects, and complete encapsulation, yielding materials with superior performance for fundamental studies. Its primary limitations are the stringent requirements for high temperature, high vacuum, or specific substrates, the associated high equipment cost and limited yield, and the frequent use of flammable/explosive organometallic precursors.

#### 2.4.2. High-Temperature Catalytic Pyrolysis: Solid-State Chemical Reconstruction

This strategy involves the pre-integration of a magnetic metal source and a carbon (or nitrogen-carbon) source into a precursor molecule or complex via chemical bonding or intimate mixing. A subsequent one-step high-temperature pyrolysis process then achieves the reduction/carburization of the magnetic metal species concurrent with the synchronous generation and integration of the graphene carbon network. Typically, a metal-organic complex or a physically mixed precursor is prepared first. High-temperature pyrolysis (e.g., 500–900 °C) is conducted under an inert atmosphere. The organic portion carbonizes and graphitizes to form graphene or doped graphene, while the metal species are reduced to metallic or carbide/oxide nanoparticles, becoming in-situ encapsulated or embedded within the forming carbon matrix. Yang et al. [86] pyrolyzed a composite of cobalt phthalocyanine (CoPc) and GO at 800 °C under argon, generating Co nanoparticle-loaded N-doped graphene, which was further oxidized to yield a Co_3_O_4_/graphene composite for use as a lithium-ion battery anode. Hu et al. [77] synthesized graphene-layer-wrapped Fe/Fe_5_C_2_ nanoparticles supported on N-doped graphene in one step by pyrolyzing a mixture of graphitic carbon nitride (g-C_3_N_4_) and ferrocene. This material exhibited oxygen reduction reaction (ORR) activity comparable to commercial Pt/C with superior stability. Research from the authors’ group developed an efficient self-catalytic pyrolysis strategy [116]. Using g-C_3_N_4_ and ferrocene as co-precursors, the iron species generated from ferrocene decomposition served not only as the magnetic source but also catalyzed the graphitization process during the pyrolysis of g-C_3_N_4_, ingeniously achieving the one-step in-situ generation of N-doped magnetic graphene. The advantages of this method include a relatively simple process, the ability to precisely control heteroatom doping via precursor design, ease of achieving carbon encapsulation, and the resultant materials’ high stability. The main limitations are high energy consumption and the potential for particle agglomeration or excessive graphitization at elevated temperatures.

#### 2.4.3. Emerging Rapid Conversion Technologies: Energy-Field-Driven Ultrafast Synthesis

These technologies utilize non-traditional, intense energy sources like electrical current (Joule heating) or electrochemical driving forces to achieve ultra-rapid conversion of precursors, offering novel, disruptive pathways for green, low-temperature, and highly efficient preparation. Ansari et al. [104] developed a concise one-step electrochemical exfoliation and deposition method. Using a graphite rod as the anode and an iron plate as the cathode in a suitable electrolyte, under an applied DC voltage, the anode graphite undergoes electrochemical oxidation and exfoliation, generating few-layer graphene. Simultaneously, the dissolving cathode provides Fe^2+^ ions, which migrate and oxidize to form Fe_3_O_4_ nanoparticles directly on the nascent graphene sheets, yielding a graphene-Fe_3_O_4_ nanocomposite in a single step. Hosseinzadeh et al. [115] reported a groundbreaking technique combining conventional and flash Joule heating. A mixture of FeCl_3_ and carbon black is first treated with conventional Joule heating to reduce its resistance. Subsequently, an instantaneous high-voltage capacitor discharge (flash Joule heating) is applied, generating ultra-high temperatures (~3000 K) for a milliseconds-long duration. This rapid pulse achieves near-instantaneous graphitization and structural transformation, converting the precursor into graphene nanostructures that exhibit room-temperature ferromagnetism due to the incorporation of atomic-level iron and defect engineering. This technology offers remarkable advantages in energy efficiency, speed, and simplicity, opening a new avenue for the macro-scale preparation of magnetic graphene and the discovery of non-equilibrium material states.

#### 2.4.4. Methodological Comparison and Synthesis Roadmap

CVD, high-temperature catalytic pyrolysis, and emerging rapid conversion technologies collectively define the cutting-edge spectrum of the Precursor Co-Conversion tier. To clarify their technological distinctions and strategic value, a systematic comparison is presented in Table 4. This comparison highlights the evolution from “vapor-phase atomic epitaxy” (CVD) to “solid-state chemical reconstruction” (Pyrolysis), and further to “energy-field-driven ultrafast reactions” (Rapid Conversion). The process complexity and demanding conditions are intrinsically linked to the pursuit of unparalleled material quality, functionality, or synthesis speed. CVD achieves near-perfect structural control at the atomic scale, serving as the indispensable “gold standard” for fundamental studies and the fabrication of model systems where ultimate structural fidelity is paramount [16]. High-temperature catalytic pyrolysis, through ingenious precursor design, masters the seamless integration of heteroatom doping with carbon encapsulation, establishing itself as the mainstream, chemistry-driven route for preparing high-performance, multifunctional materials [92,116,117]. Emerging rapid conversion technologies fundamentally overturn the spatiotemporal constraints of conventional thermal processes. Their core appeal lies in “green and rapid” synthesis, breaking barriers for scalable preparation and enabling the discovery of novel properties [104,115]. Therefore, Table 4 serves not merely as a technical catalog but as a strategic roadmap for innovation. It guides researchers in making a consequential choice based on primary objectives: select CVD for atomic-level precision when exploring intrinsic properties or constructing benchmark architectures; opt for catalytic pyrolysis for versatile functionality when tailored doping, encapsulation, and multifunctionality are the goals; and leverage emerging rapid techniques for disruptive speed and sustainability when pursuing energy-efficient, scalable production or radically new material states.

Table 4 positions Precursor Co-Conversion as the apex of synthesis sophistication, yet it is precisely here that the gap between laboratory excellence and practical viability is most pronounced. CVD, the gold standard for atomic precision, operates under conditions (>800 °C, vacuum, specialized substrates) that are fundamentally incompatible with scalable, cost-effective manufacturing. High-temperature pyrolysis offers a more pragmatic compromise, but the trade-off between graphitization degree and particle agglomeration remains poorly understood and inadequately controlled. Emerging rapid conversion technologies (flash Joule heating, electrochemical exfoliation) are genuinely exciting in their potential for ultrafast, green synthesis, but their mechanistic underpinnings are still embryonic, and the long-term stability of the resulting metastable structures is virtually unexplored. Our critical assessment suggests that the true breakthrough for this tier will not come from perfecting any single technique, but from hybridizing approaches—for instance, combining the precursor design principles of pyrolysis with the energy efficiency of flash heating. The field must also confront an uncomfortable truth: the exquisite structures produced by these methods have rarely been subjected to rigorous, application-relevant performance benchmarking against their more mundanely synthesized counterparts. Without such comparisons, the value proposition of molecular-scale precision remains largely hypothetical.

In summary, the Precursor Co-Conversion method represents the pinnacle of synthesis strategies for magnetic graphene composites, achieving integration at the molecular blueprint level. By “weaving” the components together from a common source, it enables unparalleled structural engineering across scales. While challenges in cost and scalability for some variants remain, its immense potential for creating high-performance electrocatalysts, cutting-edge electromagnetic materials, and quantum-inspired architectures is unequivocal. Future breakthroughs will hinge on a deeper mechanistic understanding of the “precursor-to-structure-property” relationship and the integration of these methods with modern manufacturing techniques (e.g., printing, patterning) for intelligent material design manufacturing techniques (e.g., printing, patterning) for intelligent material design.

## 3. Synthesis Decision Framework and Future Perspectives

Through an in-depth analysis of the four-tier evolution of synthesis strategies for magnetic graphene composites—In-situ Assembly, Single-Component In-situ Formation, Synchronous In-situ Formation, and Precursor Co-Conversion—a clear developmental trajectory from “modular combination” towards “molecular-scale integration” in precision manufacturing is evident. Each strategy, with its unique principles, addresses specific challenges regarding interface control, dispersion uniformity, and structural regulation, while simultaneously confronting its own set of scientific and engineering hurdles. This chapter aims to construct an application-oriented synthesis decision framework based on the preceding analysis and, building upon this foundation, discuss the core challenges and future directions facing the synthesis science in this field.

### 3.1. Application-Oriented Synthesis Decision Framework

The ultimate goal of material synthesis is to serve specific applications. Therefore, the choice of a synthesis strategy is fundamentally a decision-making process involving trade-offs based on target performance requirements, resource constraints, and technical complexity. Figure 3 translates our four-tier evolutionary framework into a practical decision-making tool that maps specific performance priorities to the most appropriate synthesis tier. A practical decision-making workflow can be summarized as follows:

(1) Define Core Performance Priority: First, define the material’s primary objective. Is it the pursuit of ultimate intrinsic properties (e.g., highest conductivity/thermal conductivity, strongest magnetic loss, atomic-level catalytic activity)? Or the realization of complex multifunctional integration (e.g., integrated theranostics, stimuli-responsiveness)? Or a focus on green, low-cost, large-scale production? Or perhaps the exploration of novel physical/chemical phenomena (e.g., room-temperature ferromagnetism)?

(2) Match Priority with Method Tier:

If pursuing ultimate intrinsic properties and atomic-level structural precision: Priority should be given to Tier 4: Precursor Co-Conversion, particularly CVD [16] and precise pyrolysis methods [93,118]. They can provide near-perfect crystallinity, complete carbon encapsulation, and tunable electronic structures, serving as the “gold standard” for exploring material performance limits and constructing model systems.

If requiring multifunctional integration and complex structure customization: The choice can be based on the nature of the functional modules. For systems requiring precise spatial arrangement of biomolecules, polymers, or multiple nanounits, Tier 1: In-situ Assembly (especially covalent bonding assembly [45] and polymer bridging [47]) offers maximum flexibility for modular design. For materials that require the integration of catalytic or adsorptive functions with magnetism and conductivity, the one-pot strategy of Tier 3: Synchronous In-situ Formation [9,88] is often ideal. It achieves efficient component integration and ensures strong interfacial bonding.

If the goal is economical, green, and large-scale production, the preferred choice is the co-precipitation method (Tier 2) [49,75]. This is due to its simple, aqueous-based process and ease of scale-up, making it ideal for producing large batches of adsorbents or basic catalysts. Emerging Tier 4 rapid conversion technologies (e.g., electrochemical [91], flash Joule heating [115]) also show great potential for scalability due to their efficiency and low energy consumption.

If exploring new phenomena or for rapid prototyping: Tier 4 technologies like flash Joule heating [115] can create non-equilibrium new materials at second/millisecond speeds, serving as a powerful tool for discovering new properties. The microwave-assisted method [94] in Tier 2 can rapidly screen synthesis parameters, accelerating new material development.

(3) Weigh Constraints: After matching the method tier, specific constraints must be further weighed. For example, even though CVD can provide the best performance, its high equipment cost and complex process may not be suitable for preliminary exploration or pilot production. In such cases, high-performance pyrolysis routes [116] or well-designed hydrothermal one-pot methods [9] may be a better compromise.

This decision framework shifts the focus from seeking a single “optimal” method to selecting the “most suitable” synthesis path for each unique application scenario. The transition from “trial-and-error” experience to “rational selection” based on this framework is a significant marker of maturity in synthesis science research.

### 3.2. Future Challenges and Directions: Towards a Predictive Synthesis Paradigm

**Figure 4:**
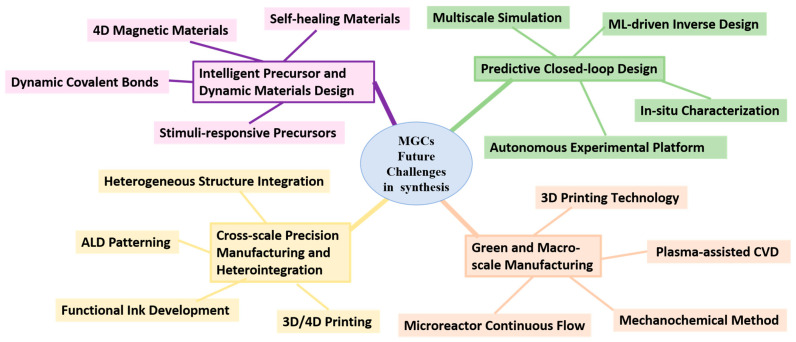
Summary of future challenges and directions for the synthesis science of MGCs. Based on critical analysis of the current state-of-the-art across all four synthesis tiers, four interconnected frontiers are identified: (1) predictive closed-loop design, (2) intelligent precursors and dynamic materials, (3) cross-scale precision manufacturing, and (4) green and macro-scale manufacturing.

Figure 4 maps the transition beyond empirical discovery toward a predictive synthesis paradigm, converging on four interdependent frontiers that collectively define the next era of precision manufacturing. The remarkable evolution of synthesis strategies has laid a solid foundation, yet the journey towards truly predictable, scalable, and intelligent manufacturing of magnetic graphene composites is just beginning. As summarized in Figure 4, future breakthroughs will converge on four interconnected frontiers that collectively define the next paradigm: moving from empirical discovery to predictive science.

#### 3.2.1. Predictive Closed-Loop Design: From Data to Discovery

The greatest limitation today is the “black-box” nature of synthesis, reliant on trial-and-error. The future lies in establishing a closed-loop design paradigm integrating operando characterization, multiscale simulation, and autonomous experimentation. The goal is to transform vast, often unstructured experimental data into predictive power—the ability to foresee synthesis outcomes and material properties. This requires building standardized, high-fidelity databases linking synthesis parameters to multiscale structural descriptors and performance metrics. Empowered by such data, machine learning (ML)-driven inverse design models can then propose optimal precursor formulations and reaction pathways for target properties. These hypotheses can be tested and refined by autonomous experimental platforms (e.g., robotic fluidic stations), creating a rapid “design-synthesis-characterization-learning” cycle that drastically accelerates discovery and optimization.

#### 3.2.2. Intelligent Precursor and Dynamic Materials Design

Future synthesis must evolve from creating static structures to programming dynamic responses. This demands a revolution in intelligent precursor design. Molecules and complexes will be engineered to be stimuli-responsive, undergoing predetermined morphological or chemical transformations upon exposure to magnetic fields, light, or specific chemical environments. Utilizing dynamic covalent chemistry, such precursors will enable the synthesis of 4D magnetic materials and self-healing composites that can adapt, reconfigure, or repair in response to operational demands. This shift from static to dynamic and adaptive material systems is essential for applications in soft robotics, reconfigurable electronics, and advanced biomedical devices.

#### 3.2.3. Cross-Scale Precision Manufacturing and Heterointegration

Advanced applications demand concurrent control over material architecture at the atomic, nano-, micro-, and macro-scale. The core challenge is the seamless integration of synthesis techniques across these disparate length scales. At the macro-scale, this involves adapting materials for 3D/4D printing and inkjet printing through the development of tailored functional inks with optimal rheological and magnetic properties. At the micro- and nano-scale, it requires integration with techniques like atomic layer deposition (ALD) for precise patterning and coating. The ultimate aim is a holistic manufacturing workflow where atomic-level doping, nanoscale heterostructure formation (e.g., core-shell), and macroscopic device geometry are all digitally designed and fabricated in an integrated manner, enabling the creation of previously unimaginable multifunctional architectures.

#### 3.2.4. Green and Macro-Scale Manufacturing for Real-World Impact

For laboratory breakthroughs to achieve societal impact, sustainable and scalable processes are non-negotiable. The focus must shift towards green macro-scale manufacturing. This entails replacing energy-intensive batch processes with continuous, efficient systems such as microreactor-assisted continuous flow synthesis, offering superior control and scalability. Simultaneously, novel low-energy activation methods like plasma-assisted CVD and solvent-free mechanochemical synthesis must be developed to dramatically reduce the environmental footprint. The ultimate success criterion is translating lab-scale precision to green, macro-scale manufacturing. This requires the ability to produce kilogram quantities with consistent quality, using benign solvents and low energy inputs, all while maintaining the exquisite structural control first demonstrated in milligram-scale syntheses.

The challenges outlined above are profound and interdependent. Overcoming them requires dismantling traditional disciplinary silos and fostering deep convergence between synthetic chemists, process engineers, data scientists, and roboticists. A critical first step is community-wide adoption of standardized data reporting for synthesis and characterization. By collectively embracing the integrated roadmap depicted in Figure 4, the field can transition decisively from an artisanal practice to a rigorous, predictive science of precision synthesis. This is the essential pathway to unlock the full transformative potential of magnetic graphene composites for energy, electronics, environmental sustainability, and human health.

## 4. Structural Regulation Strategies and Application Overview

The preceding section established a sophisticated framework for the synthesis of magnetic graphene composites, charting an evolutionary path from modular assembly to molecular-scale integration and culminating in a rational decision-making tool for selecting the optimal synthesis strategy. This deep understanding of “how to make” the material is a fundamental cornerstone. However, it raises the next pivotal question in the design sequence: how do we translate this meticulously engineered, as-synthesized microstructure into the superior, application-specific performance that motivated its creation in the first place? The answer lies in the deliberate and rational engineering of the material’s microstructure itself, which acts as the indispensable transducer between synthetic potential and functional reality.

### 4.1. Structural Engineering: The Transducer from Synthesis to Application

**Figure 5:**
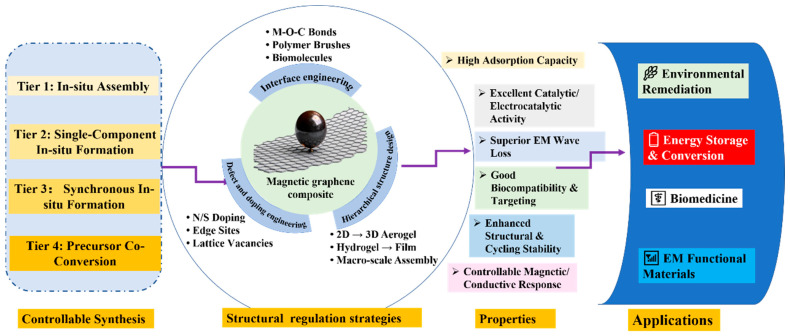
The overarching “Synthesis-Structure-Property-Application” rational design paradigm for MGCs. This diagram crystallizes the core thesis of this review: that microstructure acts as the pivotal “transducer” between synthetic potential and functional realization, integrating the synthesis framework (Section 2) and structural regulation strategies (Section 5) into a unified blueprint.

Controllable synthesis provides the material with its fundamental potential, but translating this potential into superior functionalities requires rational design of the microstructure. As schematically captured in Figure 5, the microstructure acts as a pivotal “transducer” between synthesis and application, converting synthetic possibilities into targeted performance outcomes.

As illustrated in Figure 5, the macroscopic performance of magnetic graphene composites is governed by their microstructure. This microstructure functions as a set of precise “control knobs”, serving as a bridge connecting synthesis science with practical applications. Through three core strategies, structural design translates the synthetic potential established in the previous section into targeted functionalities: interface engineering (for establishing robust interfaces and introducing stimuli-responsiveness), defect and doping engineering (for tailoring electronic and surface properties), and hierarchical structure construction (for optimizing mass transport and mechanical integrity). Through the deliberate adjustment of these structural parameters, the inherent potential of the composite is unlocked, yielding a series of outstanding performance outputs—high adsorption/catalytic activity, excellent electromagnetic loss, good biocompatibility and targeting, and enhanced electrochemical stability. These capabilities collectively underpin breakthroughs in the four major application fields (Table 5).

Table 5, while comprehensive, exposes several uncomfortable truths about the current state of the field. First, the saturation magnetization (Ms) values reported across ostensibly similar materials span an astonishing range (from ~1.4 to >200 emu/g), suggesting that factors beyond composition—such as particle size, crystallinity, surface oxidation, and measurement protocols—exert a dominant influence that is rarely systematically accounted for. Second, the temporal distribution of references reveals a concerning concentration in the 2011–2026 period, with a notable scarcity of very recent (2023–2026) entries in several sub-categories, particularly in environmental adsorption. This may indicate either a saturation of the field or a shift of research attention toward more fashionable topics. Third, and most critically, the table highlights a pervasive lack of standardization in performance reporting: adsorption capacities are measured under wildly different conditions (pH, temperature, initial concentration), making cross-study comparisons essentially qualitative. We argue that the field’s next major advance will require community-wide adoption of standardized testing protocols and reporting formats, without which the wealth of data in tables like this remains underutilized for guiding rational material design.

### 4.2. Magnetic Components: Rational Selection and Design of Functional Building Blocks

Having established the pivotal role of microstructure as the governing “control knob” for performance, the rational design of magnetic graphene composites must begin with the deliberate engineering of its most fundamental functional units. As the core of the functional binary system, the magnetic component, in concert with the graphene matrix, sets the foundational ceiling for the composite’s properties. Therefore, this section will first elucidate the application-driven logic for the rational selection and design of the magnetic component, which precedes and informs the subsequent engineering of the graphene matrix and the more advanced interfacial or hierarchical architectures discussed later.

**Figure 6:**
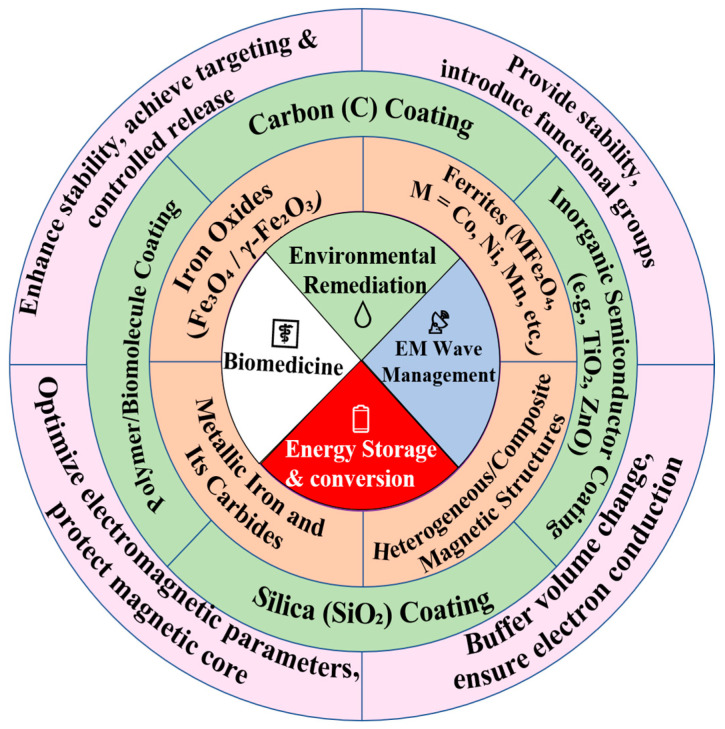
Application-driven logic for the rational selection and design of the magnetic component in MGCs. Starting from the core performance requirements of the target application domain (outer ring), the diagram guides the selection of the optimal magnetic core (middle ring) and the most suitable surface coating/modification strategy (inner ring).

The rational selection and engineering of the magnetic component constitute the first and pivotal decision in the design of high-performance magnetic graphene composites. This choice, far from arbitrary, follows an application-driven logic that begins with the core performance requirements of the target field. The magnetic component is selected not arbitrarily but application-pulled; Figure 6 formalizes this demand-driven selection logic by aligning target domain to optimal core class to required surface/coating strategy. As visually mapped in Figure 6, this decision-making paradigm starts from the outer ring representing the four major application domains (Environmental Remediation, Biomedicine, EM Wave Management, Energy Storage and Conversion). Each domain dictates a specific set of functional needs, which in turn guides the selection of the optimal magnetic core (e.g., Fe_3_O_4_/γ-Fe_2_O_3_, Ferrites, Metals/Carbides, or Heterogeneous Structures) from the middle ring. The final and critical step is the strategic application of a functional coating or modification (inner ring), such as Carbon (C) coating for stability, Polymer/SiO_2_ layers for biocompatibility and targeting, or inorganic semiconductors for catalytic activity. This sequential, demand-oriented selection process ensures that the magnetic component is not merely a passive additive but the active, functional heart of the composite, predefining its stability, interfacial properties, and core performance ceiling. The following sections will dissect each element of this paradigm, detailing the intrinsic properties of different magnetic materials and analyzing how targeted surface engineering unlocks their full potential to meet the stringent demands of advanced applications.

#### 4.2.1. Statistical Overview of Magnetic Core Types and Their Intrinsic Properties

Drawing on the data compiled in Table 5, the magnetic materials employed can be categorized into four primary classes, whose intrinsic properties constitute the functional foundation:

Iron Oxides (Fe_3_O_4_/γ-Fe_2_O_3_): This is the most widely used magnetic core, appearing across all Tiers (1–4). Their high saturation magnetization (Ms values in Table 5 commonly range from 30–70 emu/g) ensures the material possesses strong magnetic responsiveness for rapid separation. Concurrently, their good biocompatibility makes them the preferred choice for the biomedical field (e.g., targeted drug delivery [42,43,59], magnetic hyperthermia [112]) and the environmental adsorption field (e.g., removal of heavy metals [48,77], dyes [78,124]). Fe_3_O_4_ is also commonly used as an anode active material for lithium-ion batteries [7,27,101].

Ferrites (MFe_2_O_4_, M = Co, Ni, Mn, etc.): The characteristic of these materials is their high magnetocrystalline anisotropy constant, which can provide stronger magnetic loss mechanisms. Among them, CoFe_2_O_4_ is the most common and serves as a key component for constructing high-performance electromagnetic wave absorption and shielding materials [49,93,114]. Other ferrites such as NiFe_2_O_4_ [113] and MnFe_2_O_4_ [85] are used for specific adsorption or catalysis based on their unique ionic properties.

Metallic Iron and Its Carbides (Fe, Fe_3_C, Fe_5_C_2_): These materials are primarily synthesized via Tier 4 precursor co-conversion methods, often forming carbon-coated core-shell structures (e.g., Fe_3_C@C [102]). They typically possess extremely high saturation magnetization and potential high catalytic activity, mainly targeting high-end applications such as efficient electrocatalysis [92], high-performance EMI shielding [97], and advanced energy storage [102].

Heterogeneous/Composite Magnetic Structures: To pursue performance synergy, heterogeneous structures combining different materials have emerged. Examples include the core-shell structure Fe_3_O_4_@ZnO [111], which aims to combine magnetic and catalytic properties, and the heterogeneous nanospheres Cu/Fe_3_O_4_ [49], which enhance electromagnetic wave absorption by introducing interfacial polarization.

#### 4.2.2. Surface Modification and Coating Engineering: Active Design from Stabilization to Functionalization

Once the magnetic core is selected, whether and how to perform surface modification is a crucial step connecting intrinsic properties to target performance. As shown in Table 6, the main coating/modification strategies and their functional objectives are as follows:

Carbon (C) Coating: This is the core strategy for enhancing chemical stability and conductivity. The continuous carbon layer formed via CVD [31] or pyrolysis [92,116] acts as an inert barrier, protecting the magnetic core (especially Fe, Fe_3_O_4_) from oxidation or acid etching, while simultaneously forming an efficient conductive network with the graphene matrix. This is crucial for lithium-ion battery anodes [7,27] and electrocatalysis [92] operating in harsh environments, effectively buffering volume expansion and preventing deactivation.

Silica (SiO_2)_ Coating: This strategy primarily provides chemical inert isolation and a surface functionalization platform. The SiO_2_ layer effectively shields the magnetic core from direct contact with the medium, enhancing its chemical stability. More importantly, its surface-rich silanol groups (-SiOH) facilitate the grafting of various functional groups (e.g., amino, carboxyl, targeting molecules) via silanization reactions. For instance, the Fe_3_O_4_@SiO_2_ structure serves as a universal foundation for constructing complex core-shell structures (e.g., Fe_3_O_4_@SiO_2_@TiO_2_-Co [51]) and for bioconjugation [104], widely used in catalysis, biomedical separation, and detection.

Polymer/Biomolecule Coating: This strategy directly serves colloidal stability, biocompatibility, and intelligent responsiveness. Coating with polyethylene glycol (PEG) prolongs blood circulation time; using chitosan (CS) [42,47] or polydopamine (PDA) [104] improves dispersibility and biocompatibility and provides active sites for loading drugs or functional molecules; introducing pH- or reduction-sensitive polymer chains enables the construction of stimuli-responsive drug release systems [47].

Inorganic Semiconductor Coating (e.g., TiO_2_, ZnO): Aims to construct heterojunctions with the magnetic core or graphene, serving applications requiring efficient charge separation such as photocatalysis [95,111] and photoelectric conversion.

In summary, the final performance of the material results from the synergistic effect of the intrinsic properties of the magnetic core and surface engineering. Rational design must follow a clear structure-property relationship (Table 5): In the environmental and biomedical fields, Fe_3_O_4_ is mainstream due to its balanced performance, often supplemented with polymer/SiO_2_ coatings for functionalization and stabilization. In the electromagnetic wave management field, ferrites with high anisotropy (e.g., CoFe_2_O_4_) or metals/carbides with high Ms (e.g., Fe_3_C) are preferred, and the thickness and properties of their surface dielectric layer (e.g., carbon, SiO_2_) can be precisely tuned to regulate impedance matching. In energy and harsh chemical environments, any highly active magnetic core must be combined with a “armor” like carbon coating to balance activity and stability. Therefore, the design of magnetic graphene composites is a multi-dimensional optimization process. The magnetic core determines the upper limit of performance capability and the main direction of function, while surface modification and coating engineering are the precise keys to unlocking and enhancing these capabilities while compensating for their shortcomings (e.g., instability). Advancing from “functional combination” to “performance synergy” and “systematic intelligence” must be built upon a profound understanding and rational design of the complete chain: “magnetic core-surface structure-functional performance”.

Table 6 distills a clear pattern: the magnetic core defines the performance ceiling, while surface engineering determines whether that ceiling is reachable. Yet this table also reveals a curious asymmetry in research effort. While carbon coating for energy applications and polymer/SiO_2_ coating for biomedical applications are well-developed, the surface engineering strategies for electromagnetic wave management remain surprisingly primitive—often relying on trial-and-error tuning of shell thickness rather than rational design guided by electromagnetic simulation. We also note that the “one core, one coating” paradigm dominates, whereas nature routinely employs multi-layer, functionally graded interfacial designs. The field has much to learn from biological systems (e.g., nacre, bone) where hierarchical interfaces enable combinations of properties that are mutually exclusive in synthetic materials. A particularly underexplored direction is the use of stimuli-responsive coatings that can dynamically adjust their properties (e.g., dielectric constant, hydrophilicity) in response to operational conditions, enabling adaptive rather than static performance.

## 5. Structural Regulation Strategies: From Functional Combination to Performance Synergy

**Figure 7:**
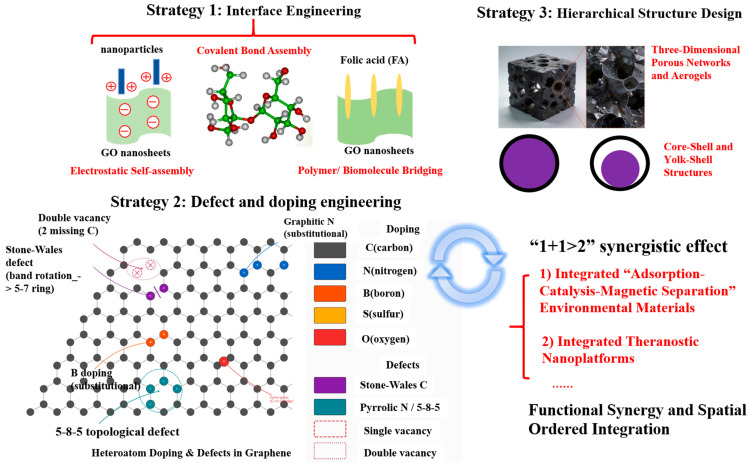
Schematic illustration of the three core structural regulation strategies for MGCs: interface engineering, defect and doping engineering, and hierarchical structure construction. These strategies collectively constitute a synergistic “toolbox” for precision performance tailoring, enabling the paradigm shift from “functional combination” to “performance synergy”.

The macroscopic properties of MGCs are fundamentally rooted in their nanoscale interfaces, defects, and electronic structures. Moving beyond simple component mixing, advanced structural regulation strategies are key to achieving targeted performance optimization. Figure 7 integrates the three core paradigms—interface engineering, defect and doping engineering, and hierarchical structure construction—showing how they operate as synergistic “control knobs” that convert the synthesis window into targeted property outcomes.

### 5.1. Interface Engineering: Constructing Robust “Bridges” and Smart “Interfacial Layers”

The interface constitutes the critical zone governing stress transfer, charge transport, energy exchange, and mass transport in composites, making the construction of a robust and functionalized interface a fundamental objective. Based on the nature of the driving forces, in-situ assembly can be mainly divided into three subcategories: electrostatic self-assembly, covalent bonding assembly, and polymer/biomolecule bridging, which exhibit significant differences in interfacial bond strength, process complexity, and functional tunability, as compared in Table 7.

Table 7 provides a convenient taxonomy of interfacial interactions, but it risks oversimplifying a fundamentally more complex reality. In practice, real composite interfaces rarely conform to a single interaction type; instead, they involve a superposition of electrostatic, covalent, hydrogen bonding, and van der Waals contributions whose relative weights depend sensitively on local chemical environment and processing history. The sharp boundaries drawn in this table are pedagogical conveniences, not physical realities. Furthermore, the table implicitly equates “stronger” with “better,” an assumption we challenge. There are applications—such as sacrificial coatings, controlled-release systems, or recyclable adsorbents—where deliberately weaker, reversible interactions are desirable. The field’s fixation on maximizing interfacial strength may be limiting the exploration of dynamic, adaptable interfaces that could enable entirely new functionalities. We suggest that future research should focus not on finding the “strongest” interface, but on designing interfaces with precisely tuned bond strength and reversibility—what might be termed “interface-by-design.”

Covalent Bond “Welding” for Enhanced Bonding: Constructing covalent bonds between functional groups on magnetic nanoparticles and graphene via chemical coupling is an effective means to achieve firm bonding and promote electron transfer. For example, Gonzalez-Rodriguez et al. [45] modified Fe_3_O_4_ with aminosilane (APTES), enabling its covalent linkage to the carboxyl groups of graphene oxide (GO) through amidation, significantly strengthening the interfacial adhesion. Similarly, in many composites for water treatment, covalent functionalization of magnetic GO (MGO) with chelating agents like EDTA [75] or polyethyleneimine (PEI) [76] introduces specific adsorption sites while simultaneously reinforcing the bonding between the modification layer and the substrate.

Polymer/Biomolecule Interfacial Layers for Multifunctionality: Introducing stimuli-responsive polymers or biomolecules as interfacial layers can simultaneously improve dispersibility, biocompatibility, and integrate new functions. Polydopamine (PDA) coating, prized for its universal adhesion and abundant functional groups, is widely used for encapsulating and bridging components (e.g., Ref. [105]). The introduction of natural polymers like chitosan (CS) or hyaluronic acid (HA) (e.g., Refs. [12,53,62]) greatly enhances the material’s biocompatibility. Their active groups enable drug loading and pH-responsive release or achieve cell targeting via receptor-ligand interactions (e.g., folic acid [47]).

### 5.2. Defect and Doping Engineering: Activating Intrinsic Activity and Precisely Tuning Electronic Structure

Intentionally introducing “imperfections” (intrinsic defects) or foreign heteroatoms into the graphene sp^2^ carbon lattice is a core lever for modulating its electronic structure, bandgap, spin properties, and surface chemical activity. Defects as Active Anchoring Sites: Creating vacancies, edges, and other defects in graphene can expose highly reactive unsaturated carbon atoms. These sites serve as powerful anchoring points, promoting the uniform and firm loading of magnetic nanoparticles (e.g., Ref. [34] anchoring Cu/Fe_3_O_4_heterospheres on defective graphene). Simultaneously, the defects themselves can act as active centers for catalysis or adsorption.

Heteroatom Doping for Performance Modulation: Doping is an effective strategy for altering the Fermi level, conductivity, and surface polarity of graphene. Nitrogen doping is the most common. Crucially, the type, concentration, and bonding configuration of dopants—which ultimately determine the electronic and catalytic properties—are intrinsically governed by the choice of synthesis method and precursor chemistry. For example, high and catalytically active pyridinic-N doping is frequently achieved through the precursor co-pyrolysis of nitrogen-rich molecules (e.g., g-C_3_N_4_, melamine) with metal salts, a hallmark of the Precursor Co-Conversion (Tier 4) strategy (Refs. [92,93]). Conversely, self-catalytic pyrolysis (Refs. [114,117]) may offer a more straightforward route for bulk doping. Thus, the ‘doping design’ is, in essence, a ‘precursor and synthesis pathway design’. Nitrogen atoms incorporated into the carbon lattice in different configurations (e.g., pyridinic N, graphitic N) not only significantly enhance the material’s conductivity and catalytic activity for reactions like the oxygen reduction reaction (ORR) [92], but their lone pair electrons can also hybridize with the d-orbitals of magnetic metals, potentially inducing room-temperature ferromagnetism [78] or modulating magnetic properties, offering possibilities for spintronic applications.

### 5.3. Hierarchical Structure Design: Optimizing Mass Transport, Impedance Matching, and Spatial Utility

Constructing hierarchical assemblies with complex spatial morphologies and graded pore structures is key to addressing common issues in nanomaterials such as easy agglomeration, low utilization of specific surface area, and limited mass transport. Core-Shell and Yolk-Shell Structures: Such structures can effectively protect the magnetic core. The intermediate void or outer shell can tune electromagnetic parameters, buffer volume change, and prolong the transmission path for electromagnetic waves or ions. These highly uniform and precisely encapsulated core-shell architectures are predominantly synthesized via advanced Tier 4 methods, particularly Precursor Co-Conversion. For instance, “pitaya-like” Fe_3_O_4_@C/rGO [57], carbon-doped ZnCo_2_O_4_ yolk-shell microspheres [96], and Fe_3_C@N-doped graphene yolk-shell structures [97] all optimize impedance matching and multiple internal reflections through their unique “core@void@shell” design, leading to outstanding electromagnetic wave absorption performance. The successful construction of these sophisticated yolk-shell (core@void@shell) geometries, which are pivotal for introducing additional interfacial polarization and regulating electromagnetic parameters, relies heavily on precisely controlled synthesis pathways. These often involve template-assisted methods or the careful design of heterogeneous precursors that undergo differential decomposition rates—strategies that fall within the high-precision realm of Synchronous In-situ Formation (Tier 3) and Precursor Co-Conversion (Tier 4) discussed in Section 2.

Three-Dimensional Porous Networks and Aerogels: Assembling two-dimensional graphene sheets into three-dimensional interconnected porous networks (e.g., foams, aerogels) can effectively suppress sheet restacking, maintain high specific surface area, and provide rapid diffusion channels for reactants, ions, or electromagnetic waves. For example, structures like 3D porous graphene foam/Fe_3_O_4_ [100] and CoFe_2_O_4_/N-rGO aerogel [115] exhibit performance superior to that of 2D stacked structures in adsorption, electromagnetic shielding, and energy storage, benefiting from their interconnected porosity and good structural integrity.

### 5.4. Functional Synergy and Spatial Ordered Integration

For complex applications like theranostics and synergistic catalysis, it is necessary to integrate multiple functional units in an orderly manner at the nanoscale to achieve a “1 + 1 > 2” synergistic effect.

Integrated Theranostic Nanoplatforms: Such designs are exemplary in biomedical applications. As shown in Refs. [30,43,53,61,62,63,65,95,108], through ingenious core-shell or multilayer structures, superparamagnetic Fe_3_O_4_ (for MRI imaging and magnetic targeting), graphene (for drug loading and photothermal conversion), fluorescent probes, functional polymers (PEGylation, targeting molecules), and therapeutic agents, are integrated into a single platform. This achieves the integration of disease diagnosis, treatment, and real-time monitoring.

Integrated “Adsorption-Catalysis-Magnetic Separation” Environmental Materials: In environmental remediation, materials are often designed to possess both adsorption-enrichment and catalytic degradation functions, coupled with easy magnetic recovery. For example, magnetic graphene loaded with CdFe_2_O_4_ [95], CoFe_2_O_4_ [107], or constructed with Z-scheme heterojunctions (Fe_3_O_4_@SiO_2_@TiO_2_-Co/rGO [51]) can not only adsorb pollutants but also degrade them photocatalytically, ultimately, enabling rapid separation via magnets. This achieves integration and greening of the treatment process.

### 5.5. Summary: The “Structure-Performance-Mechanism” Correlation

The aforementioned strategies—interface engineering, defect and doping engineering, hierarchical structure design, and functional integration—are not isolated but constitute a synergistic “toolbox” for the targeted performance optimization of magnetic graphene composites. The evolution from “functional combination” to “systematic intelligence” is microscopically rooted in the precise regulation of the “structure-performance-mechanism” relationship. This correlation can be succinctly summarized across the core application domains:

In environmental remediation, the material’s function is dictated by its surface and interface properties. High-selectivity adsorption (e.g., for Pb(II) [72]) relies on the creation of specific recognition sites through precision surface functionalization, leveraging interactions like chemical coordination or hydrogen bonding. In contrast, efficient catalytic degradation (e.g., photo-Fenton reactions [47]) depends fundamentally on constructing intimate heterojunctions or interfaces that enable efficient separation and transport of photogenerated charge carriers.

For biomedical theranostics, performance leaps are achieved through the spatial and functional ordering of multiple components. The realization of integrated “all-in-one” platforms [113] necessitates the hierarchical assembly of distinct modules (e.g., targeting ligands, imaging agents, therapeutic cargos, and stimulus-responsive linkers) into a single, spatially organized architecture, enabling synergistic targeting, imaging, and therapy.

The pursuit of broadband electromagnetic wave absorption is governed by the principles of impedance matching and synergistic loss. Achieving the “thin, lightweight, wide, and strong” ideal [49,97] is less about simply mixing dielectric and magnetic components, and more about ingeniously designing core-shell, yolk-shell, or porous 3D structures that optimize electromagnetic parameters at the macro-scale while introducing multiple polarization and scattering mechanisms at the micro/nano-scale.

Addressing the cycling stability challenge in energy storage centers on mechanical and electrochemical confinement. The primary strategy involves constructing robust carbon-based matrices or encapsulation layers (core-shell structures) that physically buffer the large volume expansion of magnetic active materials (e.g., Fe_3_O_4_) during charge/discharge cycles, while maintaining a continuous conductive network for electron transport [2].

This mechanistic understanding marks a critical transition in the field’s research paradigm: from an experience-based “trial-and-error” approach towards a “rational design” framework based on established structure-property relationships. The forward design logic is now being complemented and enhanced by a reverse design thinking: starting from the “target performance requirements,” one deduces the “necessary structural features,” and then selects the most feasible “synthesis and regulation strategies” from the available toolbox. This deep integration of mechanistic insight with material design is the essential pathway guiding the development of magnetic graphene composites from simple “functional combination” to sophisticated “performance synergy” and, ultimately, towards truly “systematic intelligent” systems.

## 6. Application Areas: Translating Structural Design into Domain-Specific Performance

The rational design principles established in preceding sections are ultimately validated by their ability to address real-world challenges. Table 8 compiles representative studies organized by our four-tier framework and application domain, linking abstract design paradigms to specific achievements. The following sections dissect each domain, demonstrating how targeted structural design overcomes performance bottlenecks.

**Figure 8:**
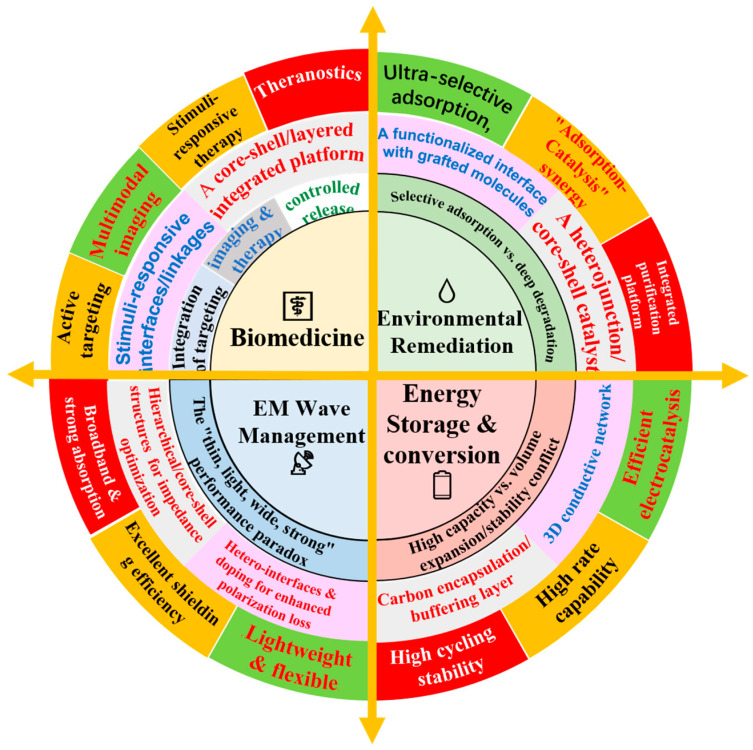
Application-domain-specific structural design paradigms for MGCs. This diagram maps the four major application domains to their core performance bottlenecks and corresponding structural solutions, demonstrating how the universal structural regulation strategies (Figure 7) are adapted to overcome unique challenges in environmental remediation, biomedicine, EMI shielding, and energy storage.

The domain-specific ways in which these structural knobs are prioritized are visually summarized in Figure 8 and then dissected in Section 6.1, Section 6.2, Section 6.3 and Section 6.4. Each of the four major domains—environmental remediation, biomedicine, electromagnetic management, and energy storage and conversion—faces distinct core challenges. To overcome these, unique structural design paradigms have been developed, leading to targeted and synergistic performance breakthroughs. The following sections will detail these structure-property-application relationships, demonstrating how the precise “tuning” of interfaces, defects, and hierarchical architectures—as outlined in Section 3—directly addresses the unique performance bottlenecks and functional imperatives of each field.

### 6.1. Environmental Remediation: Evolving from Adsorbents to Integrated Purification Systems

As underscored by cross-domain analysis, the structural design of materials for environmental remediation centers on surface chemistry and interfacial engineering to enable selective adsorption and catalytic degradation. This focus addresses a core limitation of conventional materials: the inability to seamlessly integrate high-efficiency capture, destructive degradation, and facile magnetic recovery within a single, stable platform. Precision structural engineering is the key to this integration, transforming composites from mere mixtures of components into synergistically “orchestrated” systems. As demonstrated in the following cases, this is achieved by engineering interfaces to graft molecular recognition sites, constructing heterojunctions to activate advanced oxidation pathways, and designing hierarchical architectures to optimize mass transport. Consequently, these structurally advanced composites evolve into intelligent platforms capable of executing the complete “recognition-enrichment-destruction-recovery” cycle, marking a paradigm shift from passive pollutant transfer to active, integrated purification.

#### 6.1.1. Targeted Removal of Heavy Metal Ions: Precision and Capacity

The design focus is on enhancing selectivity and capacity through surface functionalization. While basic MGO shows good adsorption, grafting specific chelating groups dramatically boosts performance. EDTA-functionalized MGO achieves high adsorption capacities of 508.4, 268.4, and 301.2 mg/g for Pb(II), Hg(II), and Cu(II), respectively [7]. For ultra-high selectivity, PVP-functionalized MGO shows a separation factor for Pb(II) over competing ions exceeding 103, enabling deep purification from 93 ppb to 0.4 ppb in real water, far below the WHO standard [72]. This strategy also enables efficient recovery of valuable or radioactive metals, as seen in polyamidoxime/PEI modified MGO which achieves a U(VI) adsorption capacity of 606.06 mg/g [119].

#### 6.1.2. Treatment of Organic Pollutants: From Enrichment to Destruction

For organics, materials are engineered for both high-efficiency adsorption and catalytic degradation. Functionalized composites achieve remarkable adsorption capacities, e.g., ~1085.3 mg/g for Rhodamine B dye [78]. Beyond enrichment, they serve as platforms for advanced oxidation processes. A representative core-shell Fe_3_O_4_@GO/MIL-101(Fe) catalyst completely degrades the pesticide diazinon within 105 min via a visible-light-driven photo-Fenton process, mineralizing 84% of the total organic carbon [40]. Similarly, CdFe_2_O_4_/graphene and similar composites act as efficient photocatalysts for dye degradation [95,107], moving towards integrated “adsorption-enrichment followed by in-situ destruction” cycles [83].

#### 6.1.3. Environmental Analysis: Enabling Trace Detection

Here, composites function as high-performance enrichment probes in analytical chemistry. Used in magnetic solid-phase extraction, they pre-concentrate trace pollutants from complex matrices. For instance, an ionic liquid-functionalized magnetic graphene adsorbent exhibits an adsorption capacity exceeding 8000 μg/g for triazine herbicides, enabling their detection at very low concentrations (0.09–0.15 ng/mL) in surface water when coupled with HPLC-MS/MS [80].

#### 6.1.4. Extended Applications: Addressing Complex Scenarios

The application scope extends to oily wastewater treatment using hydrophobic/oleophilic aerogels with high oil adsorption capacity (40–90 times their own weight) [35], and to high-end material protection. For example, pulse electrodeposited Ni-graphene coatings on permanent magnets form a dense, graphene-reinforced barrier, offering an order-of-magnitude improvement in corrosion resistance, which is crucial for long-term operation in harsh environments [89].

In summary, the trajectory in environmental remediation vividly illustrates the field’s progression, underpinned by concrete performance data. Materials have evolved from performing single-function adsorption to integrating multiple processes, and are now being designed for specific, challenging scenarios. This reflects a mature shift towards application-pulled, rational design of magnetic graphene composites, where structural engineering directly translates to quantifiable purification efficacy.

### 6.2. Biomedicine: From Passive Carriers to Intelligent Theranostic Platforms

Structural design for biomedical applications prioritizes the construction of biocompatible interfaces and stimuli-responsive, ‘intelligent’ functionalities.” The translation of magnetic graphene composites into clinically viable agents must, therefore, address fundamental challenges that simple carriers cannot: achieving precise targeting within biological complexity, seamlessly integrating diagnostic and therapeutic actions, and enabling on-demand treatment. Precision structural engineering serves as the indispensable conduit for this transformation, creating intelligent platforms via interfacial engineering for targeted delivery, hierarchical integration to orchestrate multiple therapeutic modules, and the incorporation of stimuli-responsive elements for controlled release. This architectural mastery empowers composites to navigate biological barriers and execute synergistic theranostic functions, fulfilling the promise of adaptive nanomedicine.

#### 6.2.1. Core Achievements: Quantifiable Synergy in Imaging and Therapy

The foundational breakthrough is the integration of diagnosis and treatment with measurable efficacy. A landmark PEGylated RGO-IONP nanoplatform achieved trimodal(fluorescence/MR/photoacoustic) tumor imaging in vivo. Therapeutically, guided by this precise imaging, it enabled low-power photothermal therapy (PTT) at only 0.5 W/cm^2^, resulting in complete tumor ablation and a 100% long-term survival rate (>40 days) in mouse models, a stark contrast to the control group’s median survival of 19 days [113]. Other designs integrate modalities for enhanced outcomes: a CoFe_2_O_4_/GO nanocomposite combined MRI with magnetothermal therapy and controlled drug release [61], while a magnetic-fluorescent graphene composite enabled simultaneous MRI/fluorescence imaging, PTT, and photodynamic therapy [95].

#### 6.2.2. Overcoming Biological Barriers: Targeted Delivery with Quantified Efficacy

A key indicator of “intelligence” is the ability to overcome biological barriers with measurable precision. To treat lethal brain gliomas, a composite co-modified with folic acid and transferrin achieved dual-receptor-mediated blood–brain barrier crossing. This led to a significant inhibition of tumor growth and an extension of the median survival of tumor-bearing mice from 19 days (control) to 38 days, monitored by MRI [43]. Biomimetic camouflage strategies, such as coating composites with cell membranes, further enhance in vivo targeting and circulation for improved therapeutic delivery [27].

#### 6.2.3. “On-Demand” Therapy: Stimuli-Responsive Release with Controlled Kinetics

Precision therapy is achieved by engineering materials to release drugs in response to specific tumor microenvironment (TME) cues. Using pH-sensitive hydrazone bonds, a DOX-loaded GO-based magnetic nanocomposite showed a release rate exceeding 80% within 48 h at pH 5.0 (simulating TME), but less than 20% at pH 7.4 (normal tissue), ensuring targeted action [44,96]. Similarly, GSH-responsive disulfide bonds enable intracellular-specific drug release, exploiting the high reducing potential of cancer cells [47].

#### 6.2.4. Expanded Functions: Quantified Activity Beyond Oncology

The platform’s utility extends beyond oncology. PEGylated magnetic graphene nanocomposites incorporating cobalt nanoparticles exhibited magnetically induced hyperthermia, reaching specific loss power values sufficient for antibacterial activity [29]. They also serve as dual-modal (MRI/fluorescence) imaging probes for highly sensitive cell labeling and tracking [46].

In summary, the biomedical application of magnetic graphene composites demonstrates the most vivid leap from “functional combination” to “systematic intelligence,” supported by concrete in vitro and in vivo data. Through rational integration of functional modules, they have evolved into smart systems capable of navigating biological complexity, making decisions at the disease site, and executing combined therapeutic actions with quantifiable efficacy, setting a new standard for next-generation nanomedicine.

### 6.3. Electromagnetic Wave Absorption/Shielding: The Pursuit of “Thin, Lightweight, Broadband, and Strong”

Cross-domain analysis identifies the primary task of structural design in electromagnetic functional materials as the co-optimization of heterogeneous interfaces and macroscopic hierarchical structures to achieve superior impedance matching and synergistic electromagnetic loss. The pursuit of the ideal “thin, lightweight, broadband, and strong” performance, however, presents a fundamental paradox where enhancing one attribute often compromises another. To architecturally resolve these conflicts, precision structural engineering—moving beyond simple compositional mixing—employs defect/doping engineering to tailor dielectric properties, constructs hierarchical and core-shell geometries to optimize impedance and introduce multi-scale scattering, and assembles 3D interconnected networks for lightness and integrity. This rational microstructure design enables magnetic graphene composites to circumvent traditional performance trade-offs, paving the way for advanced stealth, shielding, and telecommunication technologies.

To overcome the aforementioned challenges, representative structural solutions and performance exemplars have been developed:

(1) Addressing “Strong Absorption” and “Broad Bandwidth”: Constructing Multiple Polarization and Resonance Interfaces. Simply increasing loss often leads to impedance mismatch, preventing wave entry. Designing hetero-interfaces and defects creates abundant polarization centers within the material, enhancing loss without severely compromising impedance. For instance, anchoring Cu/Fe_3_O_4_ hetero-nanospheres on defect-rich graphene [49] leverages strong interface polarization and defect-induced dipole polarization, achieving a minimum reflection loss (RLmin) of −41.5 dB with an effective absorption bandwidth (EAB) of 5.2 GHz at a matching thickness of 2.6 mm. Similarly, constructing a Fe_3_C@N-doped graphene yolk-shell structure [97], with its unique “core@void@shell” hierarchy, effectively tunes electromagnetic parameters, achieving an RLmin of −48.2 dB at 1.5 mm thickness.

(2) Achieving “Lightweight” and Structural Stability: Assembling Three-Dimensional Interconnected Networks. The stacking of two-dimensional sheets reduces active area and increases density. Assembling components into 3D porous aerogels or foams is key to simultaneously achieving lightness, high specific surface area, and structural robustness. For example, a CoFe_2_O_4_/N-doped reduced graphene oxide aerogel [115] with a filler loading of only 20 wt% achieves an ultra-wide EAB of 6.48 GHz at 2.2 mm thickness. 3D porous graphene foam/Fe_3_O_4_ composites [100] also exhibit excellent overall performance due to their interconnected channels and integral skeleton.

(3) Exploring New Performance Dimensions: Utilizing Doping to Induce Novel Electromagnetic Properties. Moving beyond the traditional “magnetic particle + dielectric matrix” composite approach, cutting-edge research introduces magnetism into the carbon skeleton itself via heteroatom doping, opening new pathways. For instance, pure N-doped reduced graphene oxide aerogels can exhibit low-temperature ferromagnetism correlated with pyrrolic nitrogen content [115]; embedding atomic-level cobalt into the graphene lattice offers potential for spintronic devices [93]. This work exemplifies the evolution from “composite” to “intrinsic” design.

(4) Meeting Flexible and Wearable Demands: Developing Integrated Flexible Films/Composites. For flexible electronics and wearable devices, materials must combine excellent shielding/absorption efficiency with good mechanical properties. PEDOT:PSS-patched magnetic graphene films prepared by vacuum-assisted molecular patching engineering [90] form a dense conductive-magnetic loss network, achieving both high conductivity (~1085 S/cm) and exceptional shielding effectiveness (>40 dB in the X-band), making them suitable for integration into flexible devices.

In summary, the field of electromagnetic wave absorption/shielding serves as a paradigm for demonstrating how magnetic graphene composites achieve “performance leaps driven by structural design.” From the meticulously designed core-shell structures for optimized impedance matching, to the 3D networks constructed for lightweight broadband performance, and the doping engineering employed to explore new mechanisms, each performance record is underpinned by a profound understanding and precise manipulation of the material’s microstructure. This clearly validates the field’s progression from the initial stage of “functional combination” to the current era of “performance synergy” design based on a deep understanding of “structure-property” relationships.

### 6.4. Energy Storage: Constructing Stable and Efficient Electrochemical “Hearts”

Cross-domain analysis establishes that the core challenge for structural design in energy applications lies in utilizing carbon encapsulation and conductive network engineering to buffer volume expansion while simultaneously enhancing electrode stability and catalytic activity. This addresses the intrinsic duality of failure mechanisms when using high-capacity magnetic components (e.g., Fe_3_O_4_, Fe) as electrodes: severe volume changes during cycling that pulverize particles and disrupt electrical contact. Structural engineering resolves this conflict by creating intelligent confinement architectures, primarily through carbon coating (core-shell engineering) to buffer expansion and prevent aggregation and constructing 3D conductive scaffolds to accommodate strain and ensure efficient charge transport. This deliberate microstructural design transforms high-capacity components into reliably harnessed assets, bridging the gap between high energy density and long-term cycle life.

#### 6.4.1. Lithium-Ion Battery Anodes: Ingenious Strategies to Mitigate Volume Expansion

In lithium-ion batteries, the high theoretical capacity of magnetic components (e.g., Fe_3_O_4_) is counteracted by their severe volume expansion during cycling, leading to electrode pulverization and rapid capacity fade. The key innovation in magnetic graphene composites lies in constructing sophisticated buffering and confining architectures. Research has converged on several effective design paradigms: (i) Dual- or Multi-layer Encapsulation, where the active material is first wrapped by a carbon layer and then embedded within a 3D conductive graphene network, effectively mitigating mechanical stress and preventing particle aggregation [49,62]; (ii) Multidimensional Hierarchical Structures, such as sandwich-like or core-shell assemblies, which provide robust mechanical frameworks and abundant active sites while facilitating ion/electron transport [43,58]; and (iii) Integration with Green Processes and Doping, exemplified by solvent-free synthesis combined with nitrogen doping, which enhances interfacial bonding and overall electrode conductivity [61]. These structural engineering strategies collectively transform the magnetic component from a capacity contributor prone to failure into a stable, high-performance anode material, enabling high reversible capacities (often exceeding 1000 mAh/g) and significantly improved cycling stability.

#### 6.4.2. Supercapacitors and Efficient Electrocatalysis

RGO/iron carbide (Fe_3_C) nanocomposites, with their high specific surface area and good conductivity, can be used as supercapacitor electrodes, exhibiting a specific capacitance of 245 F/g at 1 A/g and demonstrating good electrochemical performance [90,102]. Reduced graphene oxide/iron carbide nanocomposites also show multifunctional potential in supercapacitor electrodes and magnetic applications [102].

In the field of electrocatalysis, graphene layer-wrapped Fe/Fe_5_C nanoparticle composites exhibit platinum-like electrocatalytic activity and outstanding stability for the oxygen reduction reaction (ORR) [92]. In alkaline media, their half-wave potential is comparable to that of commercial Pt/C catalyst (only ~30 mV negative shift), and they possess stronger methanol tolerance and superior cycling durability (negligible activity decay after 5000 cycles), offering a promising non-precious metal alternative catalyst to reduce costs for fuel cells and metal-air batteries.

## 7. Conclusions and Outlook

### 7.1. Conclusions

This review establishes a “Synthesis–Structure–Property–Application” framework for MGCs. We propose a four-tier evolutionary framework for synthesis strategies and an application-oriented decision-making tool. We systematically analyze three structural regulation paradigms—interface engineering, defect and doping engineering, and hierarchical structure construction—as synergistic “control knobs” for property tailoring. Case studies across four application domains demonstrate how targeted structural design overcomes domain-specific performance bottlenecks. This work provides a roadmap for transitioning from empirical exploration to predictive, design-driven science.

### 7.2. Outlook

Despite the substantial progress reviewed herein, several fundamental challenges must be addressed. We identify four interconnected frontiers that will shape the field’s future trajectory.

The performance–scalability paradox. Tier 4 methods enable atomically precise structures, but their high cost and harsh conditions hinder practical translation. The field must confront an uncomfortable question: has the relentless pursuit of ultimate performance inadvertently impeded industrialization? Future progress requires evaluation frameworks that explicitly weigh performance gains against manufacturability and long-term stability.

The standardization gap. Testing conditions and performance metrics vary widely across studies, making cross-comparisons largely qualitative. Without community-wide adoption of benchmark materials and reference testing protocols, thousands of individual papers risk remaining fragmented rather than accumulating into systematic knowledge.

The complexity–reliability dilemma. Stimuli-responsive and self-healing systems are appealing, but their inherent complexity raises concerns about reproducibility and long-term reliability. The field should critically assess whether “damage-tolerant” designs—which maintain performance under degradation—offer a more pragmatic path forward.

The data–reality disconnect. Machine learning holds promise for accelerating discovery, but current datasets are sparse, heterogeneous, and lack critical metadata. A prerequisite for meaningful data-driven design is a concerted community effort to build high-quality, standardized, and openly accessible databases.

Addressing these challenges requires not only technical innovation but also a cultural shift from “novelty-first” publication toward rigor, reproducibility, and cumulative knowledge building. The framework and decision-making tools provided in this review aim to support this transition.

## Figures and Tables

**Table 1:** Comparison of the four categories of synthesis methods for magnetic graphene composites.

Tier	Method Category	Core Principle	Key Advantages	Major Challenges	Representative Refs.
1	In-situ Assembly	Physical/chemical integration of pre-synthesized components.	Modularity, flexibility	Weak interface	[8,34,35,36,37,38,39,40,41,42,43,44,45,46,47]
2	Single-Component In-situ Formation	Chemical conversion and anchoring of metal ions on a GO substrate.	Enhanced dispersion, stronger bonding	Incomplete GO reduction, non-uniform growth	[9,26,27,29,30,48,49,50,51,52,53,54,55,56,57,58,59,60,61,62,63,64,65,66,67,68,69,70,71,72,73,74,75,76,77,78,79,80,81,82,83,84,85,86,87]
3	Synchronous In-situ Formation	Simultaneous reduction of GO and generation/loading of magnetic particles.	One-step integration, good interface	Complex kinetics, demanding synchronization	[7,29,88,89,90,91,92,93,94,95,96,97,98,99,100,101,102,103,104,105,106,107,108,109,110,111,112,113,114]
4	Precursor Co-Conversion	Co-transformation of integrated precursors into graphene and encapsulated magnetic species.	Atomic precision, ultimate stability	Harsh conditions, high cost, difficult scale-up	[31,90,92,103,115,116,117]

**Table 2:** Methodological comparison of in-situ assembly strategies.

Assembly Strategy	Dominant Force	Interfacial Bond Strength	Process Complexity	Material Stability	Typical Function/ Application Domain
Electrostatic Self-Assembly	Physical (Coulombic force)	Weak	Low	Environmentally sensitive	Simple composite, EM shielding [8], dye adsorption [40]
Covalent Bonding Assembly	Chemical (Covalent bond)	Strong	High	High	Biotheranostic platforms [45], advanced oxidation catalysis [118]
Polymer/Biomolecular Bridging	Combined Physical/Chemical	Medium to Strong	High	Medium to High	Targeted drug delivery [42,47], cell imaging [46], specific adsorption/separation [119]

**Table 3:** Methodological comparison of single-component in-situ formation methods.

Method Subclass	Core Driving Force & Conditions	Process Characteristics	Product Characteristics	Primary Application Directions
Hydrothermal/Solvothermal Method	High T & P (120–200 °C, autoclave)	Enables synchronous GO reduction & particle growth; Long reaction cycle.	High crystallinity & diverse morphology; Strong interfacial bonding.	High-performance EM absorption [49,57], Catalysis [50], Biomedical theranostics [44,53,122]
Co-precipitation Method	Chemical precipitation (~85 °C, alkaline, aqueous)	Simple & scalable; Particles prone to agglomeration.	Broad particle size distribution; Common adsorbent precursor.	Pollutant adsorption (heavy metals, dyes, antibiotics) [9,50,58,66,67,68,69,70,71,72,73,74,75,76,77,78,79,80,81,82,83,84,85]
Microwave-Assisted Method	Microwave radiation (Volumetric heating)	Extremely fast reaction rate; Requires specific dielectric properties.	Uniform particle size; Can form special structures.	Rapid synthesis [26], High-performance microwave absorption [94]

**Table 4:** Methodological comparison of precursor co-conversion methods.

Method Class	Core Principle & Conditions	Product Characteristics	Key Advantages	Dominant Limitations
Chemical Vapor Deposition (CVD)	Vapor-phase epitaxy High T (≈950 °C), Catalytic substrate.	Atomically precise core-shell; High crystallinity, complete encapsulation.	Unparalleled structural control & quality; “Gold standard” for model systems.	Harsh conditions (high T, vacuum); High cost, limited yield, complex setup.
High-Temp. Catalytic Pyrolysis	Solid-state reconstruction Pyrolysis of designed precursors (e.g., organometallics).	Facilitates heteroatom doping; Carbon encapsulation, monolithic integration.	Tunable via precursor chemistry; Scalable route for multifunctional materials.	High energy consumption; Risk of particle agglomeration at high T.
Emerging Rapid Conversion	Energy-field-driven ultrafast reaction (e.g., Flash Joule heating, Electrochemistry).	Unique non-equilibrium structures; Combines high conductivity & strong magnetism.	Extremely fast & energy-efficient; Enables new material states & green processing.	Mechanisms under exploration; Requires precise parameter control.

**Table 5:** A comprehensive summary of magnetic graphene composites by primary application domain.

Application Domain (Sub-Category)	Magnetic Composition	Saturation Magnetization (Ms)	Key Performance/Function	Year	Ref.
1. Environmental Remediation					
1.1 Adsorption	Fe_3_O_4_	46.6 emu/g	Adsorption of methylene blue (MB) dye	2011	[40]
	Fe_3_O_4_	39.1 emu/g	Adsorption of arsenic As(III) and As(V)	2012	[41]
	Fe_3_O_4_	~45 emu/g	Selective adsorption and separation of histidine-rich proteins	2014	[119]
	Fe_3_O_4_ QDs	18 emu/g	Adsorption of methylene blue (MB) dye	2017	[124]
	Fe_3_O_4_	18.2 emu/g	Adsorption of Cd(II) and Pb(II) heavy metal ions	2017	[48]
	Fe_3_O_4_	N/A	Efficient adsorption of Pb(II) ions	2019	[72]
	Fe_3_O_4_	28.9 emu/g	Adsorption of Pb(II), Hg(II), Cu(II) ions	2015	[75]
	Fe_3_O_4_	35.1 emu/g	Selective adsorption of fumaric acid	2019	[76]
	Fe_3_O_4_	61.9 emu/g	Adsorption of Pb(II) and crystal violet	2018	[77]
	Fe_3_O_4_	44, 18 emu/g	Adsorption of rhodamine B (RhB) dye	2019	[78]
	Fe_3_O_4_	N/A	Adsorption of tetracycline (TC) and ciprofloxacin (CIP)	2018	[79]
	Fe_3_O_4_	68.2 emu/g	Adsorption of methylene blue (MB)	2015	[105]
	CoFe_2_O_4_	40.38 emu/g	Adsorption of acid fuchsin dye	2016	[107]
1.2 Catalysis & Advanced Oxidation	Fe_3_O_4_	47.8, 30.3 emu/g	Peroxymonosulfate activation for pesticide degradation	2020	[118]
	Fe_3_O_4_@SiO_2_@TiO_2_-Co	N/A	Photocatalytic degradation of methylene blue (MB)	2019	[51]
	CdFe_2_O_4_	14.26 emu/g	Photocatalytic degradation of methylene blue (MB)	2014	[95]
	Fe_3_O_4_	19.65 emu/g	Photocatalytic degradation of methylene blue (MB)	2020	[106]
	CoFe_2_O_4_	40.38 emu/g	Photocatalytic degradation of acid fuchsin dye (Primary)	2016	[107]
	γ-Fe_2_O_3_	33.8 emu/g	Catalytic wet peroxide oxidation of organic pollutants	2019	[109]
2. Biomedicine					
2.1 Drug Delivery & Therapy	CoFe_2_O_4_	58.4 emu/g	Drug delivery, magnetic hyperthermia, T_2_ MRI contrast	2014	[12]
	γ-Fe_2_O_3_	40 emu/g	Multimodal imaging guided chemo-photothermal therapy	2018	[30]
	Fe_3_O_4_	41.78 emu/g	Targeted drug (doxorubicin) delivery and MR imaging	2017	[42]
	Fe_3_O_4_	15.98 emu/g	Chemo-photothermal therapy for glioma	2019	[43]
	MnFe_2_O_4_	110 emu/g	Drug delivery, T_2_ MRI contrast, magnetic hyperthermia	2013	[44]
	Fe_3_O_4_	45.8 emu/g	Targeted drug delivery, dual-modal (MRI/PA) imaging	2019	[45]
	Fe_3_O_4_	1.39 emu/g	pH/redox-responsive drug delivery, chemo-photothermal therapy	2018	[46]
	Fe_3_O_4_	61.1 emu/g	Targeted drug delivery, fluorescence/MR imaging, photothermal therapy	2016	[53]
	Fe_3_O_4_	48 emu/g	Targeted delivery of doxorubicin	2018	[59]
	Fe_3_O_4_	35.5 emu/g	Multifunctional platform for imaging and therapy	2018	[61]
	Fe_3_O_4_	52.3 emu/g	Targeted drug delivery, MR/fluorescence imaging, photothermal therapy	2017	[62]
	Fe_3_O_4_	45.8 emu/g	Magnetically targeted drug delivery, photothermal therapy, MRI	2012	[63]
	Fe_3_O_4_	63.4 emu/g	Drug delivery, MR/fluorescence imaging, photothermal therapy	2016	[65]
	Fe_3_O_4_	43.7 emu/g	Drug delivery, photothermal therapy, up conversion luminescence imaging	2015	[95]
	Fe_3_O_4_	61.5 emu/g	Multifunctional nanoplatform for imaging and therapy	2016	[104]
	Fe_3_O_4_	N/A	Dual-modal imaging (MRI/Fluorescence), photothermal/photodynamic therapy	2014	[108]
	Fe_3_O_4_	76.4 emu/g	Drug delivery, MR imaging, magnetic hyperthermia	2016	[110]
	Fe_3_O_4_	48.9 emu/g	Targeted drug delivery & magnetic hyperthermia	2017	[112]
	Fe_3_O_4_	64.3 emu/g	Drug delivery, magnetic hyperthermia, MR imaging	2016	[113]
2.2 Biosensing & Separation	Fe_3_O_4_	68.2 emu/g	Cell imaging, separation, and photothermal therapy	2015	[46]
	Fe_3_O_4_@ZnO	36.8 emu/g	Photocatalytic, antibacterial, and biosensing applications	2019	[111]
3. Electromagnetic Functional Materials					
3.1 Wave Absorption & Shielding	Fe_3_O_4_	N/A	High-efficiency electromagnetic interference (EMI) shielding	2020	[7]
	Cu/Fe_3_O_4_	N/A	Multi-band (C, X, Ku) electromagnetic wave absorption	2024	[49]
	CoFe_2_O_4_	45.1 emu/g	Electromagnetic wave absorption, potential in spintronics	2018	[93]
	ZnCo_2_O_4_(Yolk-Shell)	N/A	Electromagnetic wave absorption for stealth	2019	[96]
	Fe_3_C@NG	N/A	Electromagnetic wave absorption and shielding	2024	[97]
	Fe_3_O_4_	41.73 emu/g	Electromagnetic wave absorption and shielding (foam)	2018	[100]
	CoFe_2_O_4_	39.7 emu/g	Electromagnetic wave absorption	2017	[114]
	CoFe_2_O_4_	N/A	Electromagnetic wave absorption and shielding (aerogel)	2020	[115]
	FeCo alloys	215 emu/g	Electromagnetic wave absorption	2018	[116]
4. Energy Storage & Conversion					
4.1 Battery Anode	Fe_3_O_4_	N/A	Anode for lithium-ion batteries	2020	[7]
	Fe_3_O_4_@C	18.96 emu/g	Anode for lithium-ion batteries	2019	[27]
	Co_3_O_4_	N/A	Anode for lithium-ion batteries	2010	[101]
	Fe_3_C	~70 emu/g	Anode for lithium-ion batteries	2018	[102]
4.2 Electrocatalysis	Fe/Fe_5_C_2_	N/A	Oxygen reduction reaction (ORR) for fuel cells	2017	[92]
	Fe_3_O_4_/FexC	46.49 emu/g	Electromagnetic shielding, piezoresistive sensing, environmental remediation	2025	[117]
5. Fundamental & Multifunctional Studies					
5.1 Synthesis & Fundamental Properties	(Various)	N/A	Review: Synthesis and properties of magnetic nanocomposites	2017	[26]
	Fe_3_O_4_@C	84.5 emu/g	Synthesis and enhanced microwave absorption properties	2018	[31]
	Fe_3_O_4_	76.9 emu/g	Magnetically separable photocatalyst	2013	[103]
5.2 Multifunctional/Cross-Domain	Fe_3_O_4_	61.8 emu/g	Adsorption, photocatalysis, and antibacterial activity	2019	[50]
	Fe_3_O_4_/Fe_x_C	46.49 emu/g	Electromagnetic shielding, piezoresistive sensing, environmental remediation	2025	[117]

**Table 6:** Application orientation of magnetic components and surface design strategies.

Target Application Domain	Core Performance Requirements	Preferred Magnetic Core	Key Surface Design Strategy	Functional Objectives
Environmental Remediation	High magnetic response, stability, specific adsorption/catalytic sites	Fe_3_O_4_/γ-Fe_2_O_3_	Polymer/SiO_2_ coating followed by functionalization, semiconductor composite	Provide stability, introduce functional groups, construct heterojunctions
Biomedicine	Superparamagnetism, biocompatibility, targeting & responsiveness	Fe_3_O_4_/γ-Fe_2_O_3_	SiO_2_/Polymer (PEG, CS) coating, targeting molecule modification	Enhance stability, prolong circulation, achieve active targeting & controlled release
Electromagnetic Wave Management	Strong magnetic/dielectric loss, broadband impedance matching	Ferrites (e.g., CoFe_2_O_4_), Fe/Fe_3_C	Carbon/SiO_2_ coating (regulate dielectric), construct core-shell/yolk-shell structures	Optimize electromagnetic parameters, introduce interfacial polarization, protect magnetic core
Energy Storage & Conversion	High capacity, long cycle life, high conductivity	Fe_3_O_4_, Fe/Fe_3_C	Carbon coating is essential	Buffer volume change, prevent agglomeration/deactivation, ensure electron conduction

**Table 7:** Comparison of key interaction mechanisms in structural regulation.

Interaction Type	Bonding Nature	Strength	Process Complexity	Material Stability	Function/Application Example
Electrostatic Self-assembly	Physical (Coulombic force)	Weak	Low	Environmentally sensitive	Simple composites, EM shielding [8], dyeadsorption [40]
Covalent Bond Assembly	Chemical (Covalent bond)	Strong	High	High	Theranostic platforms [45], advanced oxidation catalysis [118]
Polymer/Biomolecule Bridging	Physical/Chemical combination	Medium-Strong	Medium-High	Medium-High	Targeted drug delivery [42,47], cell imaging [46] specific adsorption/separation [119]

**Table 8:** Summary of literature on magnetic graphene.

Ref. No.	Name of Magnetic Graphene Products	Framework Tier	Synthesis Method	Application Field	Pub. Year
[29]	Magnetite/Reduced Graphene Oxide Nanocomposites	3	Hydrothermal, Solvothermal, Co-precipitation-Reduction	Magnetic Solid-Phase Extraction in Environmental Analysis; Water Treatment and Environmental Remediation	2015
[88]	PEDOT:PSS-patched magnetic graphene films	3	Hydrothermal	Electromagnetic Interference Shielding, Radar Stealth, Electronic Devices	2024
[48]	Cu/Fe_3_O_4_ heterogeneous nanospheres anchoring defect-rich graphene	2	Hydrothermal	Multi-band Electromagnetic Wave Absorption (C, X, Ku bands)	2024
[26]	Magnetic interactions in graphene	2	Microwave-assisted Solvothermal	Magnetic Nanocomposites	2021
[34]	Magnetically Graphene Oxide Embedded Chitosan	1	In-situ Assembly	Photocatalytic Degradation of Dyes (e.g., Reactive Red 198, Blue 133) in Textile Wastewater	2024
[115]	Magnetic graphene nanostructures	4	Joule Heating and Flash Heating	Room-Temperature Ferromagnetic Graphene, Potential in Spintronic Devices	2025
[89]	Reduced graphene oxide/iron carbide nanocomposites	3	Intercalation-Thermal Treatment	Multifunctional Magnetic and Electrochemical Energy Storage (Supercapacitor Electrodes)	2014
[31]	Core-Shell Fe_3_C@Graphene Nanoparticles	4	Chemical Vapor Deposition (CVD)	Multifunctional Applications	2018
[90]	Magnetic Iron-Based Nanoparticles Encapsulated in Graphene/Reduced Graphene Oxide	3	Solvothermal	Biomedical Applications (Drug Delivery, Magnetic Hyperthermia)	2024
[49]	Magnetic graphene oxide nanocomposite	2	Co-precipitation	Adsorption of Heavy Metals (Cd(II)) and Dyes (Methylene Blue, Orange G) for Water Treatment	2013
[50]	Fe_3_O_4_@SiO_2_@TiO_2_-Co/rGO Magnetic Photocatalyst	2	Hydrothermal	Photocatalytic Degradation of Methylene Blue for Wastewater Treatment	2019
[91]	Graphene Layers-Wrapped Fe/Fe_3_C_2_ Nanoparticles	4	Pyrolysis	Oxygen Reduction Reaction (ORR) for Fuel Cells and Metal-Air Batteries	2017
[92]	Embedding Atomic Cobalt into Graphene Lattices	3	Pyrolysis	Graphene-based Spintronic Devices (Spin Valves, Magnetic Sensors)	
[93]	Core-Shell Structured CoFe_2_O_4_/rGO/SiO_2_ Nanocomposites	2	Microwave-assisted Chemical Reduction	Microwave Absorption for Electromagnetic Shielding (2–8 GHz)	2020
[51]	Hierarchical Sandwiched Fe_3_O_4_@C/Graphene Composite	2	Hydrothermal-Carbonization	Anode Material for Lithium-ion Batteries (High Capacity, Cycling Stability)	2019
[52]	Graphene-based magnetic composites	2	Hydrothermal	Synergistic Chemo-Photothermal Therapy of Tumor Cells: Magnetic Targeting, Drug Delivery	2019
[94]	Yolk-Shell ZnO-Ni-C/RGO Composite Materials	3	Mechanical Mixing-Annealing	Microwave Absorption for Electromagnetic Wave Absorption Materials	2017
[95]	Carbon-Doped ZnCo_2_O_4_ Yolk-Shell Microspheres Compounded with Magnetic Graphene	3	Co-precipitation-Chemical Reduction	Electromagnetic Wave Absorption, Multi-band Coverage for Stealth Technology	2019
[53]	Polyacrylamide-grafted magnetic reduced graphene oxide nanocomposite	2	Co-precipitation	Dye Wastewater Treatment in Environmental Remediation: Adsorption of Congo Red (CR) Dye	2019
[9]	PEGylation of graphene/iron oxide nanocomposite	2	Solvothermal	Cancer Therapy in Biomedicine: Magnetically Targeted Drug Delivery, Photothermal Therapy	2020
[54]	CoFe_2_O_4_/graphene magnetic nanocomposite	2	Hydrothermal	Drug Analysis in Electrochemical Sensing: Atenolol (AT) Detection	2020
[96]	Fe_3_C@N-doped graphene layers yolk-shelled nanoparticles on the graphene sheets	3	Pyrolysis	Electromagnetic Wave Absorption and Shielding	2024
[97]	Nano-dispersed iron/graphene catalyst	3	Solvothermal and in-situ Reduction	Water Treatment: Phenol Degradation	2017
[98]	Fe_3_O_4_ clusters-NG	3	Hydrothermal-Heat Treatment	Electromagnetic Wave Absorption and Shielding	
[7]	Carbon layer encapsulated Fe_3_O_4_@Reduced graphene oxide	3	Hydrothermal-Heat Treatment	Anode Material for Lithium-ion Batteries in Energy Storage	2020
[99]	3D porous magnetic graphene foam-ferrite nanocomposite	3	Hydrothermal	Electromagnetic Wave Absorption and Shielding	2018
[35]	Magnetically Recyclable Graphene Oxide	1	In-situ Assembly	Oily Wastewater Treatment in Environmental Remediation	2021
[100]	Magnetically Separable CdFe_2_O_4_/Graphene Catalyst	3	Formation–Hydrothermal	Dye Wastewater Treatment in Environmental Remediation: Photocatalytic Degradation of MB	2014
[56]	Pitaya-like Fe_3_O_4_@C magnetic microspheres on reduced graphene oxide nanosheets	2	Solvothermal	Electromagnetic Wave Absorption and Shielding	2020
[57]	Magnetic Reduced Graphene Oxide Nanocomposite	2	Co-precipitation	Mycotoxin Detoxification in Food Safety: Adsorption of Aflatoxin B1	2024
[36]	Graphene@metal oxide core-shell nanostructures	1	In-situ Assembly	Anode Material for Lithium-ion Batteries in Energy Storage	2011
[37]	Fe_3_O_4_/Nitrogen-doped Graphene Composite	1	Solid-state Shear Pan-milling	Anode Material for Lithium-ion Batteries in Energy Storage	2018
[27]	Porous microcrystalline graphene oxide supported Fe_3_O_4_@C nanoparticle composite	2	precipitation	Anode Material for Lithium-ion Batteries in Energy Storage	2017
[90]	Reduced graphene oxide/iron carbide nanocomposites	3	High-Temperature Pyrolysis	Multifunctional Energy and Magnetic Applications: Supercapacitor Electrodes and Magnetic Materials	2014
[102]	superparamagnetic reduced graphene oxide-Fe_3_O_4_ nanocomposite	3	In-situ Chemical Method	Targeted Cancer Therapy in Biomedicine: Drug Loading and Release	2020
[103]	Magnetic Graphene-Fe_3_O_4_ Nanocomposite	3	Electrochemical Exfoliation	Multifunctional Nanotechnology Applications	2020
[104]	Polydopamine interface encapsulating graphene and immobilizing ultra-small, active Fe_3_O_4_ nanoparticles	3	Co-precipitation	Dye Wastewater Treatment in Environmental Remediation: Adsorption of Methylene Blue (MB) Dye	2021
[105]	Reduced graphene oxide/Fe_3_O_4_ hybrid nanocomposites	3	Solvothermal	Dye Wastewater Treatment in Environmental Remediation: Photocatalytic Degradation of Methylene Blue (MB)	2020
[38]	Magnetite nanoparticle decorated reduced graphene oxide	1	In-situ Assembly	Dye Wastewater Treatment (Crystal Violet, CV) and Antifungal Activity in Environmental Remediation and Biomedicine	2020
[8]	Flexible Fe_3_O_4_/graphene foam/polydimethylsiloxane composite	1	In-situ Assembly	High-Efficiency Electromagnetic Interference Shielding	2020
[39]	Magnetic graphene	1	In-situ Assembly	Water Sample Pretreatment in Environmental Analysis: Enrichment of Sulfonamide Antibiotics	2011
[58]	magnetite/graphene oxide nanocomposites	2	Co-precipitation	Targeted Drug Delivery in Biomedicine: Doxorubicin Hydrochloride (DOX) for Cancer Therapy	2018
[106]	Magnetic CoFe_2_O_4_/graphene nanocomposites	3	Hydrothermal	Dye Wastewater Treatment in Environmental Remediation: Photocatalytic Degradation of Methylene Blue	2015
[40]	Fe_3_O_4_ nanoparticles-graphene oxide	1	In-situ Assembly	Dye Wastewater Treatment in Environmental Remediation: Adsorption of Methylene Blue and Neutral Red	2010
[41]	Magnetic graphene oxide composites	1	In-situ Assembly	Heavy Metal Wastewater Treatment in Environmental Remediation: Pb(II) and Cd(II) Adsorption	2020
[59]	Graphene nanosheets/magnetite hybrid material	2	Solvothermal	Pollutant Adsorption and Wastewater Treatment; Biomedical Carrier	2011
[60]	Cobalt ferrite/graphene oxide nanocomposites	2	Co-precipitation	Theranostics in Biomedicine: Synergistic Function of Magnetic Resonance Imaging and Controlled Drug Delivery	2016
[61]	Multifunctional chitosan magnetic-graphene (CMG) nanoparticles	2	Solvothermal	Cancer Theranostics in Biomedicine: Synergistic Targeted Chemo-Gene Therapy and MRI Real-Time Monitoring	2013
[42]	Biocompatible nanocomposite of graphene oxide and magnetic nanoparticles	1	In-situ Assembly	Drug Delivery and Imaging in Biomedicine: Drug Loading and Release	2017
[44]	Graphene oxide-based magnetic nanocomposites	1	In-situ Assembly	Drug Delivery (Melittin) for Cancer Therapy: Loading, Controlled Release, Magnetic Targeting	2020
[45]	Multifunctional graphene oxide iron oxide nanoparticles	1	In-situ Assembly	Magnetically Targeted Drug Delivery, Dual-Modal Imaging (MRI and Fluorescence), Cancer Sensing	2019
[62]	Functionalized Graphene Oxide-Iron Oxide Nanocomposite	2	Solvothermal	Magnetically Targeted Drug Delivery, Photothermal Therapy, Magnetic Resonance Imaging for Cancer Theranostics	2012
[46]	Biocompatible dendrimer-functionalized graphene oxide	1	Glutathione (GSH) Bridging of Fe_3_O_4_ and GO-G4	Cell Imaging, Fluorescent Labeling for Biomedical Research	2012
[9]	PEGylation of graphene iron oxide nanocomposite	2	Solvothermal	Doxorubicin Release, Magnetically Targeted Drug Delivery and Photothermal Therapy for Cancer Treatment	2020
[107]	Magnetic and fluorescent graphene	3	Microwave-assisted Decomposition Reduction	Dual-Modal Imaging (MRI and Fluorescence), Photothermal and Photodynamic Therapy for Cancer Theranostics	2014
[47]	Magnetic graphene oxide	1	In-situ Assembly	Targeted Drug Delivery, Multi-stimuli Responsive (pH, Reduction, Magnetic) for Cancer Treatment	2017
[64]	Magnetic Graphene	2	Solvothermal	MRI Imaging, Dual Targeting (Magnetic and Receptor-Mediated), Chemo-Photothermal Combined Therapy for Glioma Treatment	2014
[30]	Graphene-based magnetic-responsive hybrids	2	Co-precipitation	Cancer Theranostics: Multi-modal Imaging (MRI, Fluorescence, Photothermal) Guided Chemo-Photothermal Synergistic Therapy	2018
[65]	magnetic graphene oxide nanomaterial	2	Co-precipitation	Adsorption and Co-adsorption of Heavy Metal Ions and Organic Pollutants (e.g., Tetracycline) for Water Treatment	2018
[66]	Polyamine modified magnetic graphene oxide	2	Co-precipitation	Dye Adsorption (Methyl Violet and Acid Red 88) for Textile Wastewater Treatment	2019
[67]	magnetic graphene oxide composite chitosan beads	2	Co-precipitation	Removal of Heavy Metals Ni(II) and Organic Dye Reactive Blue 19 (RB19) from Water	2019
[68]	magnetic graphene oxide composite nanoribbons	2	Solvothermal	Radioactive Wastewater Treatment in Nuclear and Environmental Engineering: Adsorption of Thorium (Th(IV))	2018
[69]	Carboxymethyl cellulose supported magnetic graphene oxide composites	2	Co-precipitation	Radioactive Wastewater Treatment in Environmental Remediation: Adsorptive Removal of Uranium (U(VI))	2019
[119]	Polyamidoamine/Polyethyleneimine Magnetic Graphene Oxide	1	In-situ Assembly	Radioactive Wastewater Treatment in Environmental Remediation: Efficient Removal of Uranium (U(VI)) from Mine Radioactive Wastewater	2019
[118]	Core-shell structured magnetic graphene oxide@MIL-101(Fe)	1	In-situ Assembly	Advanced Oxidation Water Treatment in Environmental Remediation: Degradation of Pesticides Diazinon (DIZ) and Atrazine (ATZ)	2020
[109]	Magnetic graphene composite	3	One-pot Method	Sample Pretreatment in Environmental Analysis: Adsorption of Triazine Herbicides (e.g., Cyanazine, Ametryn, Atrazine)	2017
[70]	Magnetic Graphene Oxide	2	Co-precipitation	Multi-purification in Water Treatment: Heavy Metal Adsorption, Microbial Disinfection	2020
[71]	Polyvinylpyrrolidone functionalized magnetic graphene-based composites	2	Co-precipitation	Heavy Metal Wastewater Treatment in Environmental Remediation: Efficient Adsorption of Pb(II)	2019
[72]	Graphene quantum dot decorated magnetic graphene oxide	2	Co-precipitation	Dye Wastewater Treatment in Environmental Remediation: Adsorption of Cationic Dyes (Methylene Blue MB and Rhodamine B RhB)	2020
[73]	Three-dimensional magnetic graphene oxide foam/Fe_3_O_4_ nanocomposite	2	Co-precipitation	Heavy Metal Wastewater Treatment in Environmental Remediation: Adsorption of Cr(VI)	2014
[74]	EDTA functionalized magnetic graphene oxide	2	Co-precipitation	Heavy Metal Wastewater Treatment in Environmental Remediation: Adsorption of Pb(II), Hg(II) and Cu(II)	2015
[75]	Polyethylenimine modified magnetic graphene oxide nanocomposites	2	Co-precipitation	Heavy Metal Wastewater Treatment in Environmental Remediation: Adsorption of Cu^2+^	2015
[10]	Magnetic Graphene Oxide Grafted Polymaleicamide Dendrimer Nanohybrids	2	Co-precipitation	Heavy Metal Wastewater Treatment in Environmental Remediation: Adsorption of Pb(II)	2017
[110]	Magnetic three-dimensional graphene/chitosan/nickel ferrite nanocomposite	3	Hydrothermal	Heavy Metal Wastewater Treatment in Environmental Remediation: Adsorption of Pb(II)	2018
[76]	Poly(β-cyclodextrin)-conjugated magnetic graphene oxide	2	Hydrothermal	Synergistic Adsorption of Heavy Metals and Organic Pollutants in Environmental Remediation: Adsorption of Cd^2+^ and Sulfamethazine (SMT)	2019
[77]	EDTA and chitosan functionalized magnetic graphene oxide	2	Solvothermal	Dye Wastewater Treatment in Environmental Remediation: Adsorption of RhB Dye	2019
[78]	magnetic graphene oxide/diethylenetriaminepentaacetic acid nanocomposite	2	Co-precipitation	Antibiotic Wastewater Treatment in Environmental Remediation: Adsorption of Tetracycline (TC) and Ciprofloxacin (CIP)	2018
[79]	Novel magnetic multi-templates molecularly imprinted polymer	2	Co-precipitation	Detection and Removal of Organic Pollutants in Environmental Remediation: Adsorption of Alkylphenols	2018
[80]	Magnetic Graphene Oxide	2	Salt Reduction	Dye Wastewater Treatment in Environmental Remediation: Adsorption of Azo Dye Eriochrome Black T (EBT)	2017
[81]	magnetic graphene oxide composites	2	Solvothermal	Wastewater Treatment in Environmental Remediation: Adsorption of Cu^2+^, Methylene Blue	2018
[82]	Magnetic graphene oxide	2	Co-precipitation	Pesticide Residue Detection and Removal in Environmental Analysis: Adsorption of Triazole Pesticides	2018
[83]	magnetic iron oxide/graphene oxide nanocomposites	2	Co-precipitation	Organic Pollutant Wastewater Treatment in Environmental Remediation: Adsorption of Dyes and Phenolic Pollutants	2017
[84]	magnetic graphene nanomaterials	2	Co-precipitation–Thermal/Chemical Reduction	Organic Pollutant Remediation in Environmental Remediation: Adsorption of Phenanthrene	2019
[85]	Graphene Oxide-Wrapped Magnetite Nanoclusters	2	Solvothermal	Dye Wastewater Treatment in Environmental Remediation: Adsorption of Methylene Blue (MB), Rhodamine B (RhB) and Methyl Orange (MO)	2018
[86]	Graphene oxide decorated with MnFe_2_O_4_ magnetic nanoparticles	2	Solvothermal	Heavy Metal Wastewater Treatment in Environmental Remediation: Pb(II) Adsorption	2018
[122]	Magnetic polymer aerogel	1	In-situ Assembly	Dye Wastewater Treatment in Environmental Remediation: Adsorption of Malachite Green (MG) Dye	2019
[111]	Magnetic Fe_3_O_4_@ZnO@graphene oxide nanocomposite	1	In-situ Assembly–Chemical Precipitation	Dye Wastewater Treatment in Environmental Remediation: Photodegradation of Methyl Orange (MO)	2019
[112]	Functionalized Graphene Nanosheets Anchored with Magnetic Nanoparticles	3	Hydrothermal	Cancer Theranostics in Biomedicine: Multimodal Tumor Imaging, Photothermal Tumor Therapy	2012
[116]	Nitrogen-Doped Magnetic Graphene	4	Self-catalytic Pyrolysis	Electromagnetic Shielding, Piezoresistive Sensing, Environmental Remediation	2025
[117]	Magnetic N-doped graphene composite	4	Self-catalytic Pyrolysis	Antibiotic Wastewater Treatment in Environmental Remediation: Photocatalytic Degradation of Tetracycline	2025
[113]	Magnetic graphene-based nanocomposites	3	Solvothermal-Heat Treatment	Heavy Metal Wastewater Treatment in Environmental Remediation: Cr(VI) Adsorption	2020
[114]	CoFe_2_O_4_/N-doped reduced graphene oxide aerogels	3	Solvothermal-Freeze Drying	Electromagnetic Wave Absorption and Shielding	2020
[103]	Fe_3_O_4_/graphene oxide nanocomposites	1	In-situ Assembly	Magnetic Solid-Phase Extraction (MSPE) Adsorbent, Enrichment and Detection	2012
[87]	Magnetite/porous graphene-based nanocomposites	2	Solvothermal-Heat Treatment	Wastewater Treatment in Environmental Remediation: Adsorption of Methyl Violet (MV), Electrosorption of Pb^2+^, Cu^2+^ and Cd^2+^ ions	2017
[124]	Fe_3_O_4_ quantum dots-graphene nanocomposites	1	In-situ Assembly–Hydrothermal	Dye Wastewater Treatment in Environmental Remediation: Adsorption of MB Dye	2017

## Data Availability

No new data were created or analyzed in this study. Data sharing is not applicable to this article.
